# Shared decision making using digital twins in knee osteoarthritis care: a randomized clinical trial of an AI-enabled decision aid versus education alone on decision quality, physical function, and user experience

**DOI:** 10.1016/j.eclinm.2025.103545

**Published:** 2025-10-04

**Authors:** Prakash Jayakumar, Paul J. Rathouz, Eugenia Lin, Zoe Trutner, Lauren M. Uhler, John Andrawis, Karl M. Koenig, Joel Tsevat, Kevin J. Bozic

**Affiliations:** a Department of Surgery and Perioperative Care, Dell Medical School at the University of Texas at Austin, Austin, TX, USA; b Department of Population Health, The University of Texas at Austin, Austin, TX, USA; c Department of Orthopaedic Surgery, The Mayo Clinic, Phoenix, AZ, USA; d Department of Orthopaedic Surgery, The University of Utah, Salt Lake City, UT, USA; e Department of Orthopedic Surgery, Harbor-UCLA Medical Center, Torrance, CA, USA; f ReACH Center and Department of Medicine, Long School of Medicine, The University of Texas at San Antonio, San Antonio, TX, USA

**Keywords:** Patient centric clinical decision support, Patient reported outcome measurement, Shared decision making, Digital twins: artificial intelligence, Machine learning

## Abstract

**Background:**

Patient decision aids (DAs) improve decision quality during shared decision-making (SDM) for patients seeking care for knee osteoarthritis (OA). However, few DAs incorporate the ‘digital twin’ concept where comprehensive data are applied to computational models to generate dynamic virtual simulations and predictions to augment decision-making in real-time. We developed an artificial intelligence-enabled DA (AI-DA) that generated digital twins using patient reported outcome measurements (PROMs) and clinical data to enhance SDM by providing personalized predictions of risks and benefits for patients with knee OA considering total knee arthroplasty (TKA). We assessed the impact of the AI-DA on patient- and process-level outcomes.

**Methods:**

We performed a randomized open-label clinical trial at a single, university-affiliated orthopaedic clinic in the USA involving patients with knee OA between February 2021 and November 2022. Patients received a full AI-DA incorporating patient education, preference assessment, and person-specific benefit:risk predictions of TKA (intervention group) or patient education only (control group). Outcomes included the Knee OA Decision Quality Instrument (K-DQI) (primary outcome), CollaboRATE SDM survey, Decision Conflict Scale (DCS), Decision Regret Scale (DRS), and Knee Injury and Osteoarthritis Outcome Score Joint Replacement (KOOS JR) for knee-specific health at 3 and 6 months post-randomization, satisfaction, appointment duration, and TKA rates. This study is registered with ClinicalTrials.gov, NCT04805554.

**Findings:**

The analytic sample comprised 101 patients ([mean [SD], 64.9 [10.1] years; 54 [54%] women]) in the intervention group and 100 patients (mean [SD] age 63.4 [8] years; 60 [60%] women) in the control group. The intervention group reported higher decision quality (mean [SD] K-DQI: 84.4 [25.2] versus 71.4 [29.8], *P* = 0.0011), lower decision conflict (DCS: 1.0 [3.1] versus 3.3 [5.8], *P* = 0.0029), lower decision regret at 6–9 months (DRS: 18.2 [19.5] versus 27.2 [24.2], *P* = 0.0051), better knee-specific health (KOOS JR: 69.5 [17.3] versus 47 [18.4], *P* < 0.0001) at 6–9 months, and greater treatment concordance (91% versus 76%, *P* = 0.0043). SDM scores, knee health at 3 months, patient and clinician satisfaction, appointment duration, TKA rates, and decision regret at 3 months were similar between groups.

**Interpretation:**

AI-DAs provide a more personalized, data-augmented SDM experience that can improve decision quality and longer-term health-related outcomes in patients with knee OA considering TKA.

**Funding:**

10.13039/100000133Agency for Healthcare Research and Quality Grant (R21HS027037).

**Research in context**

**Evidence before this study**

Knee osteoarthritis (OA) is a leading cause of knee pain and physical functional limitation worldwide. Total knee arthroplasty (TKA) for knee OA is one of the most frequently performed surgeries in the US. While many patients demonstrate substantial improvements in physical function and symptom intensity following TKA, approximately 20% do not achieve clinically meaningful improvement and remain dissatisfied with longer-term outcomes. Evidence supports the feasibility and utility of patient decision aids (DAs) to facilitate shared decision-making (SDM) in the management of knee OA. However, widespread uptake of DAs in routine clinical practice is low. There is growing interest in a more personalized approach to SDM using patient reported outcome measurements (PROMs) to increase DA adoption in orthopaedic care. Further, there is rapidly emerging interest in the use of artificial intelligence (AI) to generate person specific predictions of surgical outcomes using PROM data to better inform SDM in patients with knee OA considering TKA. Studies assessing the clinical effectiveness of AI-enabled DAs (AI-DAs) concerning aspects related to decision making (decision quality, choice awareness, collaborative decision making, and deliberation), alongside the longer-term impact on PROMs are lacking.

We searched PubMed, from database inception to September 2025, for papers published with no language restrictions, using the terms “patient outcomes”, “patient reported outcome measurements”, “patient-centric clinical decision support”, “shared decision making”, “artificial intelligence”, “machine learning”, “osteoarthritis”, and “arthritis”. Our search yielded 20 results.

**Added value of this study**

Patients experience the novel AI-DA, consisting of patient education, preference assessment, and person-specific benefit-risk predictions using computational models creating virtual ‘digital twin’ simulations for individual patients, had higher decision quality scores, less decision conflict and decision regret, better knee-specific health status, similar satisfaction, and similar TKA rates after 6–9 months compared with those receiving educational content alone. Improved decision quality and health-related outcomes can be achieved using AI-DAs that generate ‘digital twins’ to provide a more personalized, data-driven approach to SDM.

**Implications of all the available evidence**

Integrating AI-DAs within routine orthopaedic care pathways may increase the adoption of PROMs and SDM to enable a more personalized, data-augmented approach to treatment selection aligned with a patient’s preferences, values, and needs. These tools can better inform patients and surgeons during treatment decision making, drive appropriate selection of TKA, and improve outcomes, expectations, and experiences for patients seeking care for knee OA.

## Introduction

Knee osteoarthritis (OA) represents a substantial proportion of the global burden of disease, with cases projected to increase by 75% from 2020 to 2050.^1^ Recent systematic reviews underscore the need for personalized treatment plans to manage this condition.^2^ Care for knee OA is preference-sensitive and discretionary as multiple treatment options exist, including total knee arthroplasty (TKA)—one of the most frequently performed operations in the US.^3^ While many patients demonstrate substantial improvements in physical function and symptom intensity following TKA,^4^ studies show that approximately 20% do not achieve clinically meaningful improvement and remain dissatisfied with longer-term outcomes.^5^^,^^6^ Failure to meet expectations in such a substantial proportion of patients has underscored the need for improving the selection of patients most likely to benefit from TKA.^7^^,^^8^

Shared decision-making (SDM) provides a framework for enhancing clinician-patient conversations related to treatment selection and care planning. Patient decision aids (DAs) are tools designed to facilitate SDM via the transfer of knowledge regarding the condition, treatment options, and associated benefits and risks so patients can make informed treatment selections aligned with their preferences, values, and goals.^9^ Several DAs have been developed for patients considering TKA for knee OA with earlier studies indicating improvements in decision quality and patient satisfaction when using these tools.^10^ However, a recent systematic review described a dearth of high quality clinical trials and inconsistent evidence supporting their utilization and efficacy.^11^^,^^12^

Patient reported outcome measurements (PROMs) are the current gold standard in validated instruments designed to quantify health status from the patient’s perspective and incorporate the patient voice during SDM. For instance, levels of symptom intensity and physical function captured by PROMs can guide richer clinician-patient discussions, and help track and improve health outcomes during care delivery.^13^ Further, evaluating change in PROM scores over time and comparing these to established thresholds denoting specific levels of clinical benefit may augment the decision making process.^14^^,^^15^ While the literature underscores the potential for patient-centric clinical decision support using PROMs, their universal adoption in clinical practice remains low. Similarly, clinical integration of DAs and DAs incorporating PROMs to enhance SDM is also lacking.

We sought to better understand whether generating ‘digital twins’ to provide a more personalized, data-informed, and action-oriented approach to SDM using PROM data could close the knowledge-implementation gap and present a stronger use case for these tools during routine clinical practice. The ‘digital twin’ concept is defined as a virtual representation using comprehensive data that can be dynamically updated to augment decision-making in real-time through precise simulations and predictions.^16^ Such representations are usually achieved via computational models including those driven by artificial intelligence (AI) and machine learning (ML)—technology capable of synthesizing complex relationships and performing predictive analytics utilizing large data sets. Applying AI/ML to PROM data representing aspects of knee health can generate personalized predictions of clinically meaningful health outcomes, including the likelihood of improvement in pain and physical function following TKA for knee OA.^17^^,^^18^ Incorporating person-specific predictive analytics within DAs and demonstrating efficacy may drive greater adoption of such tools at the point-of-care.

Our overarching goal was to evaluate an AI-enabled DA (AI-DA) (Joint Insights, OM1, Boston, USA) incorporating patient education, preference assessment, and person-specific predictions of benefits, risks, and relevant clinical outcomes generated using population-level demographic, clinical, and PROM data. The primary objective of our study was to assess the impact of the AI-DA compared to patient education only on decision quality. Secondarily, we evaluated levels of decision conflict, decision regret, shared decision making, physical function at early and medium to longer term follow-up, alongside treatment concordance, patient and clinician satisfaction, patient-clinician consultation duration, and TKA rates between both groups.

## Methods

### Study design

We performed a single-site randomized open-label clinical trial (RCT) to evaluate the AI-DA at the Musculoskeletal Institute, Dell Medical School at the University of Texas at Austin, Austin, TX. USA. The Musculoskeletal Institute clinic provides a comprehensive multidisciplinary team-based approach to OA management offering non-operative strategies including physical therapy, home exercise programs, guided self-management, behavioral therapies, social support, and lifestyle coaching (nutritional guidance, sleep hygiene, weight management), all within a co-located facility, alongside surgery at a partner hospital or ambulatory surgical center. A research fellow attended a pre-clinic briefing (the morning huddle) at which the care of all patients scheduled for clinic that day was discussed. The lead orthopaedic surgeons for the clinic (KJB, KMK) and research fellows (EL, ZT) identified eligible patients. We obtained informed, digital consent via the Research Electronic Data Capture (REDCap) system. Consenting patients were subsequently randomized to intervention or control group. The local Institutional Review Board at Dell Medical School, The University of Texas at Austin, reviewed and provided ethical approval for this study (Approval no. 2018110042). The Consolidated Standards of Reporting Trials (CONSORT) reporting guideline were followed (see Supplement 1, Trial protocol).

### Participants

Between February 2021 and November 2022, we approached 222 patients with a presumptive diagnosis of knee OA and candidacy for primary TKA. Inclusion criteria were age 45–89 years, fluency in English or Spanish, a primary diagnosis of advanced knee OA (determined by radiographic Kellgren–Lawrence [KL] grade 3 or 4, where grade 0 represents no OA and grade 4 severe OA), ability to provide informed consent, medical fitness for TKA, a body mass index (BMI) of 20–46 kg/m^2^, and Knee Injury and Osteoarthritis Outcome Score for Joint Replacement (KOOS JR) between 0 and 85 (possible range: 0 [total knee disability] to 100 [perfect knee health]).^19^ The limits for age, BMI, and KOOS JR were set based on threshold requirements for the computational model within the AI-DA. Patients with knee problems due to other conditions (e.g., trauma, inflammatory arthropathy); advanced OA affecting other lower extremity joints requiring care first; any prior lower extremity total joint arthroplasty involving the hip, knee, or ankle; or prior musculoskeletal care for OA by an orthopaedic specialist were excluded. We periodically assessed race/ethnicity and insurance status within cohorts across both clinics to ensure each was broadly representative of the diverse patient population and not skewed toward a specific group.

### Randomization and masking

The randomization sequence was prepared by an independent statistician and loaded into the randomization module in the REDCap system. Consenting patients were randomized to intervention or control group via 8 strata based on cross-classification of 3 binary variables: ethnicity (Latino versus non-Latino), insurance status (medical access program [MAP] insurance [a plan available for underserved communities] versus non-MAP) and orthopaedic surgeon (KJB versus KMK), and, within stratum, in randomly sequenced blocks of 4 or 6, to which study team, except statisticians were blinded. This stratification and blocking ensured balance of these 3 variables between intervention and control groups over time and within stratum. Neither provider nor study participant knew or could guess the next allocation in the sequence until the participant was consented, and the intervention began, at which time, due to the nature of the intervention (i.e., needing to know grouping assignment ahead of the SDM discussion), patients, researchers and clinicians were no longer blinded to treatment arm.

### Procedures

**Fig. 1:**
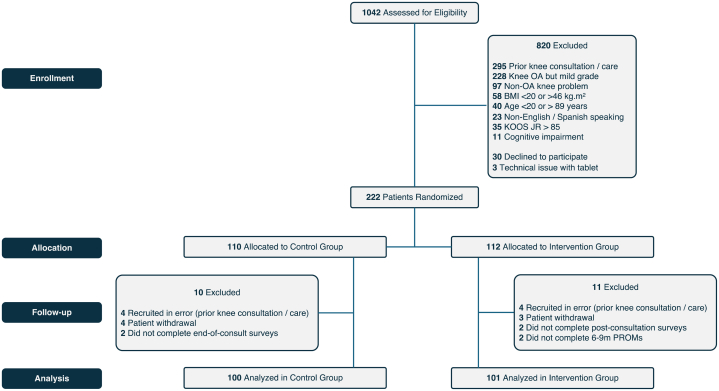
CONSORT flow diagram. BMI, Body Mass Index (calculated as weight in kilograms divided by height in meters squared); KOOS JR, Knee Injury and Osteoarthritis Outcome Severity Score Joint Reconstruction; OA, osteoarthritis; PROMs, Patient Reported Outcome Measurements.

Patients randomized to the intervention group received the complete AI-DA consisting of a patient education module, a preference assessment module, and a person-specific TKA outcome prediction module with a personalized report, or to a control group that received the patient education module alone (Fig. 1). Individuals in both groups were provided all services as needed per usual standard of care.

The patient education module provides diagrammatic and written content on OA and the condition being a natural part of aging, the potential for accommodation of the condition, alongside the risks, benefits, and clinical efficacy of evidence-based non-operative and operative treatment options, set at a 6th–7th-grade reading level. The module also includes a 4-question test on the covered material.

The interactive preference assessment module includes 3 questions focused on patient preferences regarding: 1) desired level of lasting pain relief; 2) readiness to take time off for postoperative recovery; and 3) level of acceptance of surgical risk. Each of these items are scored on a rating scale anchored with “surgical management” on one end and “non-surgical management” on the other. An additional 3 questions enquired whether patients felt they: 1) knew enough about the risks and benefits of surgery; 2) were aware of what was most important to them; and 3) felt they could make the right treatment decision.

The person-specific prediction module utilizes an AI/ML algorithm trained on a nationally representative sample of over 700,000 patients who underwent TKA. The algorithm incorporates a supervised data model utilizing clinical and outcomes data for these individuals and employs a combination of regression and gradient boosting frameworks trained for stratified prediction of the estimated probabilities of benefits from TKA surgery. Benefits for the predictive algorithm development were defined as achieving the minimal clinically important difference in KOOS JR scores alongside an improvement in domains of the KOOS JR (knee stiffness, overall knee pain, and quality of life) versus no change or worsening of outcomes. The algorithm is also trained to determine an estimated complication rate based on a specific set of adverse events (Fig. 1). Sociodemographic, lifestyle, clinical, and patient-reported outcome (PRO) variables from registry and health system data across the US were used to train the model including age, sex, smoking status, BMI, clinical comorbidities, KL grade, level of physical function (KOOS JR)^19^ and mental health (PROMIS Global Mental Health component),^20^ and number of emergency department visits and hospitalizations in the prior year. The model predicting benefits demonstrated good discriminative performance, with an area under the receiver operating characteristic curve (AUROC) of 0.77, as measured on an independent validation dataset. The model predicting overall risk of complications achieved an AUROC of 0.72, also evaluated on an independent validation set. A summary of responses to knowledge questions from the education module and patient preferences within the preferences module are provided alongside the predictive analytics in the report. All module content was made available in English or Spanish.

The research fellow ensured those randomized to the control group received patient education, and those randomized to the intervention group received patient education, preference assessment, and the person-specific report with sufficient time to review it before proceeding with the SDM consultation.

### Outcomes

**Fig. 2:**
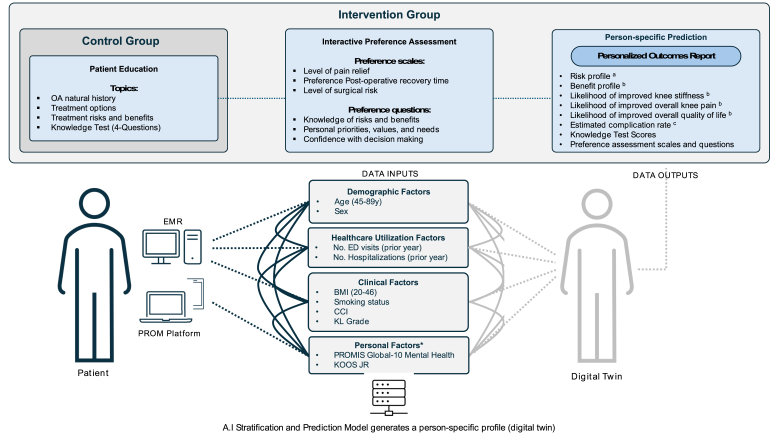
Schematic of the Artificial Intelligence-enabled Decision Aid (AI-DA) including exposure of the AI-DA components to control and intervention groups, and factors used to generate person-specific prediction outputs. ^a^Risk defined as the likelihood of experiencing no change in condition or being worse off after undergoing surgery. ^b^Complication rate defined as estimated complication rate due to joint infection within 90 days, pulmonary embolism or death within 30 days, and pneumonia, sepsis, or acute myocardial infarction within 7 days. ^c^Benefit defined as inverse of risk prediction; Likelihood of experiencing at least a minimal clinically important difference in functional outcome based on knee stiffness, knee pain, and quality of life sub-domains of KOOSJR.

We collected pre-consultation baseline data including patient demographics, clinical factors, process-level factors, and patient factors (Fig. 2). Patient age, sex, BMI, smoking status, Charlson Comorbidity Index (CCI), KL grade for knee OA, number of emergency department visits and inpatient hospitalizations in the previous year, PROMIS Global-10 Mental Health component score,^20^ and KOOS JR^19^ were data inputs used to inform the prediction from the AI/ML model for participants in our study (Fig. 1).

We assessed outcome measures post-consultation and at 3- and 6–9-months follow-up (Fig. 2). The Knee OA Decision Quality Instrument (K-DQI) ‘SDM decision process scale’ scores from questions 3.1 through 3.5 summed, formed the primary outcome including key aspects of decision quality.^21^

Secondary outcome measures included level of shared decision making (CollaboRATE survey)^22^; Decisional Conflict Scale 10-item version (DCS)^23^; Decision Regret Scale (DRS) at 3 months and 6–9 months follow-up^24^; KOOS JR^19^ at 3 months and 6–9 months follow-up (including relative to baseline KOOS JR); patient satisfaction with the consultation with the orthopaedic surgeon (Numerical Rating Scale, NRS-P); clinician satisfaction with the consultation (NRS-C); and both TKA rate and treatment concordance based on the K-DQI Question 1.6 assessed at 9 months follow-up of the last recruited patient to ensure sufficient time for patients selecting TKA to have their procedure scheduled and completed.^21^ We prioritized capture of decision process scores and treatment concordance scores from the K-DQI to represent quality of the decision-making process. We excluded the knowledge scoring component of this survey as items tended to focus on the surgical procedure rather than reinforcing the benefits offered by evidence based non-operative strategies alongside the potential to accommodate the condition. Moreover, both intervention and control groups received patient education where extensive knowledge testing (19 questions versus 4 questions) would substantially increase response burden.

We captured demographic, clinical, process, and patient factors directly from the patient and transferred these data into our REDCap database and cross-checked information with the electronic medical record (Athena, Boston, MA, USA). PROM data were captured by in-person or telephone assessment.

### Statistical analysis

We performed descriptive statistics to describe the study population using frequencies and/or means and standard deviations organized by study arm. For formal comparison between arms on study endpoints, we compared the groups based on means or relative frequencies. For binary endpoints, we tested significance with Fisher’s Exact Test; for ordinal endpoints with fewer than 10 categories, we employed the Armitage Trend test, while for continuous endpoints, we used non-parametric rank sum tests. Two endpoints (DRS and KOOS JR) were assessed longitudinally at 3 and 6–9 months. We analyzed these measures under a linear mixed effects model with a random intercept at the subject level, and treatment-by-time interaction, yielding separate treatment effects at each of 3 and 6–9 months. For KOOS JR, we adjusted for the baseline value, thereby increasing statistical power by reducing the error variance in the regression equation. Based on the 13 combined primary and secondary outcomes, we assessed for significance using *P*-values after multiple comparison adjustments according to the Hocherg (1988) approach. We executed formal analyses in Stata v.18.

We calculated sample size for by assuming we would treat the Decision Process score of the DQI as continuous. We aimed to detect a treatment effect size (i.e., Cohen’s *D*) as small as 0.5, with a Type I error rate of 0.05 and power of 0.90, assuming equal sample size in intervention and control arms. Given 8 randomization strata, we estimated a needed sample size of 180 participants (90/group). With an estimated loss-to-follow-up rate of 10%, we aimed to enroll 200 participants. This study is registered with ClinicalTrials.gov, NCT04805554.

### Role of the funding source

The study sponsor had no role in study data collection, analysis, and interpretation of data; in the writing of the report; or in the decision to submit the paper for publication.

## Results

**Fig. 3:**
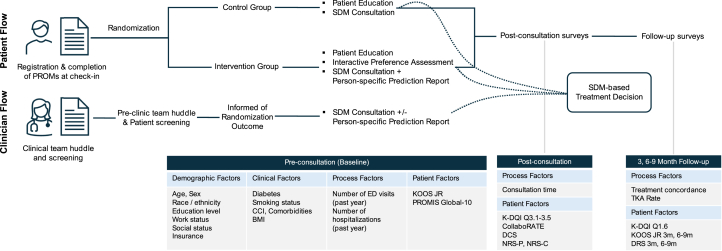
Schematic of patient and clinician flow including demographic, clinical, process, and patient factors captured at baseline, post-consultation, and at 3 month and 6–9 month follow-up. BMI, Body Mass Index (calculated as weight in kilograms divided by height in meters squared); CCI, Charlson Comorbidity Index (based on Anaemia (preoperative), Cardiac arrythmia, Electrolyte disorder, Peripheral vascular disease, Valvular disease, Urinary tract infection, Unintended weight loss, Chronic pulmonary disease, Congestive heart failure, Ischaemic heart disease, Peptic ulcer disease, Cardiovascular disease, Hypothyroidism, Dementia); DCS, Decision Conflict Survey; DRS, Decision Regret Survey; ED, emergency department; K-DQI, Knee Decision Quality Instrument; KOOS JR, Knee Injury and Osteoarthritis Outcome Severity Score Joint Reconstruction; NRS-C, Numerical Rating Scale for Satisfaction with Care (Clinician); NRS-P, Numerical Rating Scale for Satisfaction with Care (Patient); PROMIS Global-10, Patient Reported Outcome Measurement Instrumentation System Global 10; SDM, Shared Decision Making.

We analyzed data from a final total of 201 patients (101 patients in the intervention group (mean [SD] age 64.9 [10.1] years; 54 [53%] women); 100 patients in the control group (mean [SD] age 63.4 [8] years; 60 [60%] women; Tables 1 and 2 and Fig. 3). None of the randomized patients moved between the two study groups. Data were analyzed based on the original assigned groups (intent to treat paradigm). All analyses, including primary endpoints and secondary endpoints were pre-specified. Analyses were adjusted for the baseline value where baseline values were present for an endpoint. Notably, there were no adverse events, serious adverse events, or unintended effects in either cohort.

**Table 1:** Descriptive statistics of patient demographic and other characteristics by study group.

	Participants, No. (%) or mean (SD)	
	Full tool (n = 101)	Education only (n = 100)
Age, mean (SD), years	64.9 (10.1)	63.4 (8.0)
Women	54 (53)	60 (60)
**Race/Ethnicity**		
White/Caucasian	32 (32)	37 (37)
Asian	2 (2)	2 (2)
Black or African American	15 (15)	10 (10)
Hispanic or Latino	51 (50)	50 (50)
Other	1 (1)	1 (1)
**Education**		
High school or less	58 (57)	47 (47)
College	15 (15)	39 (39)
Advanced degree	28 (28)	14 (14)
**Work status**		
Unemployed	34 (34)	31 (31)
Disability support	3 (3)	2 (2)
Employed	34 (33)	44 (44)
Retired	30 (30)	23 (23)
**Social status**		
Living alone	22 (22)	21 (21)
Partnered	79 (78)	78 (78)
Part-time care^a^	0	1 (1)
**Insurance**		
MAP/Medicaid	52 (52)	55 (55)
Other	49 (48)	45 (45)
**Health history**		
Diabetes	15 (15)	22 (22)
Smoking status		
Current	9 (9)	6 (6)
Former	21 (21)	31 (31)
Never	71 (70)	63 (63)
CCI, mean (SD)	0.55 (0.91)	0.63 (0.97)
BMI, mean (SD)	32.6 (6.3)	33.1 (6.5)
**Baseline PROMs**		
PROMIS global 10 physical, mean (SD)	40.1 (8.9)	38.3 (8.9)
Mental, mean (SD)	47.9 (10.9)	46.9 (9.6)
**Baseline knee health**		
KOOS-JR, mean (SD)	37.9 (18.3)	36.5 (20.2)
Duration of pain, mean (SD), months	61.1 (57.4)	68.8 (104.1)

Notes: Continuous variables presented as mean (standard deviation); categorical variables presented as percentages.

Abbreviations: MAP, Medical Access Program for underserved population in Central Texas; CCI, Charlson Comorbidity Index; BMI, body mass index; PROMIS, Patient Reported Outcome Measurement Information System; KOOS JR, Knee Injury and Osteoarthritis Outcome Score Joint Reconstruction.

a Patients live alone but occasionally have some form of help.

**Table 2:** A comparisons of primary and secondary endpoints for the AI-DA (intervention) versus patient education alone (control).

	Randomization Arm		Delta or OR (95% CI)^c^	*P*-value^d^^,^^e^
	Intervention	Control		
	Mean (SD)/f (%)	Mean (SD)/f (%)		
Total N	**101**	**100**		
**Decision making impact**				
K-DQI - process score	84.4 (25.2)	71.4 (29.8)	13.0 (5,2, 20.3)	**0.001**
Process score				
0	1 (1.0%)	2 (2.0%)		
20	7 (6.9%)	11 (11.0%)		
40	3 (3.0%)	14 (14.0%)		
60	10 (9.9%)	13 (13.0%)		
80	17 (16.8%)	21 (21.0%)		
100	63 (62.4%)	39 (39.0%)		
Decision conflict score	1.0 (3.1)	3.3 (5.8)	−2.3 (−3.7, −0.9)	**0.003**
CollaboRATE score	25.6 (3.9)	25.0 (3.6)	0.6 (−0.5, 1.6)	0.122
Below maximum, No. (%)	32 (31.7%)	41 (41.0%)	0.7 (0.37, 1.2)	0.189
Decision regret score				
3 months	22.4 (20.0)	25.9 (20.4)	−3.4 (−9.8, 2.9)	0.289
6–9 months	18.2 (19.5)	27.2 (24.2)	−9.0 (−15.4, −2.7)	**0.005**
**Knee-specific health impact**				
KOOS JR				
3 months	51.4 (20.1)	48.3 (17.4)	3.1^a^ 2.0 (−3.0, 6.9)	0.433
6–9 months	69.5 (17.3)	47.0 (18.4)	22.5^a^ 22.3 (17.4, 27.1)	**<0.001**
KOOS JR MCID^b^ at 6–9 Months	72 (74.2%)	37 (38.5%)		
KOOS JR SCB^b^ at 6–9 Months	61 (62.9%)	26 (27.1%)		
KOOS JR PASS^b^ at 6–9 Months	44 (45.4%)	12 (12.5%)		
**Experience impact**				
Patient satisfaction (NRS-P)	9.7 (1.2)	9.5 (1.1)	0.2 (−0.1, 0.5)	0.201
Clinician satisfaction (NRS-C)	9.2 (1.1)	9.1 (1.3)	0.2 (−0.2, 0.5)	0.362
**Process impact**				
Consult duration (min)	24.0 (10.7)	22.5 (10.6)	1.5 (−1.8, 4.2)	0.358
TKA desired	24 (23.8%)	19 (19.0%)	1.9 (0.92, 3.8)	0.106
TKA proportion	25 (24.8%)	15 (15.0%)		0.111
Treatment concordance	92 (91.1%)	76 (76.0%)	3.2 (1.4, 7.4)	**0.004**

Statistical analysis performed using STATA. StataCorp. 2023. Stata Statistical Software: Release 18. College Station, TX: StataCorp LLC. Key routines: “nptrend”, “tab2 with exact test”, “xtmixed”, “bootstrap”, “ranksum”, “collect”.

Abbreviations: K-DQI, Knee Osteoarthritis Decision Quality Instrument; KOOS JR, Knee Injury and Osteoarthritis Outcome Score Joint Replacement; TKA, total knee arthroplasty.

*P*-values in bold represent statistical significance.

a The first entry is the unadjusted difference of means. The second entry is adjusted within mixed models for baseline values.

b MCID set at a 6-month minus baseline difference >15; SCB a difference >20, PASS is an absolute 6-month score >70.

c Summary statistics shown are mean (standard deviation, SD) for continuous variables, or frequency (%) for categorical variables. Confidence Intervals (CIs) for mean differences (Delta) based on BCa bootstrap CI’s with 1000 repetitions^25^ (except for longitudinal mixed models which were sufficiently normally distributed to use Wald test from mixed models). CI’s for odds ratios (for binary endpoints) are based on Woolf’s method.

d Hypothesis tests were implemented as follows: Binary endpoints (Below CollaboRATE maximum; KOOS JR MCID, SCB, PASS; TKA desired; TKA proportion; Treatment concordance): Fisher’s Exact test. Ordinal endpoints (K-DQI Process Score; Patient satisfaction; Clinician satisfaction) (defined here as <10 unique values): Armitage Trend Test. Continuous endpoints (DCS; CollaboRATE; Consult duration): non-parametric rank-sum test. Longitudinal analysis of the DRS and KOOS-JR: Wald test in linear mixed model with random effect at the subject level and treatment-by-time interactions. KOOS-JR at 3 and 6 months was adjusted for the baseline value. Categorical variables presented as percentages.

e All five *P*-values < 0.05 are significant after multiple comparison adjustments according to Hochberg approach.^26^

### Primary outcome

**Fig. 4:**
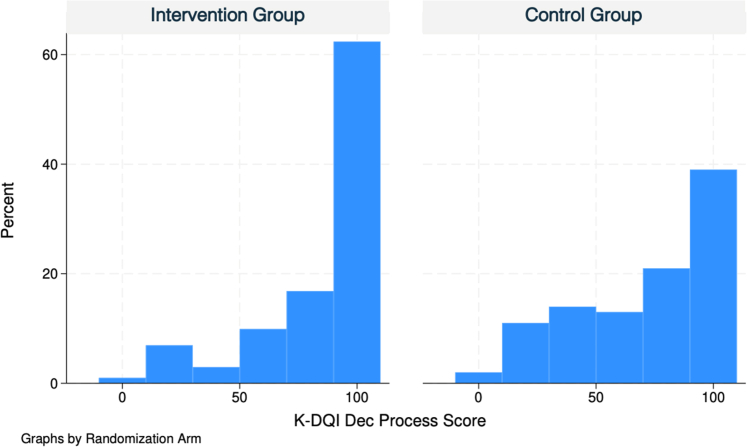
Histogram showing distribution of decision quality scores using the Knee Decision Quality Instrument (K-DQI) process score.

Patients in the intervention group had higher mean [SD] K-DQI decision quality process scores than the control group (84.4 [25.2] versus 71.4 [29.8], *P* = 0.0011); Delta = 13.0 ([95% CI: 5.2, 20.3]) (Table 2, Fig. 4).

### Secondary outcomes

**Fig. 5:**
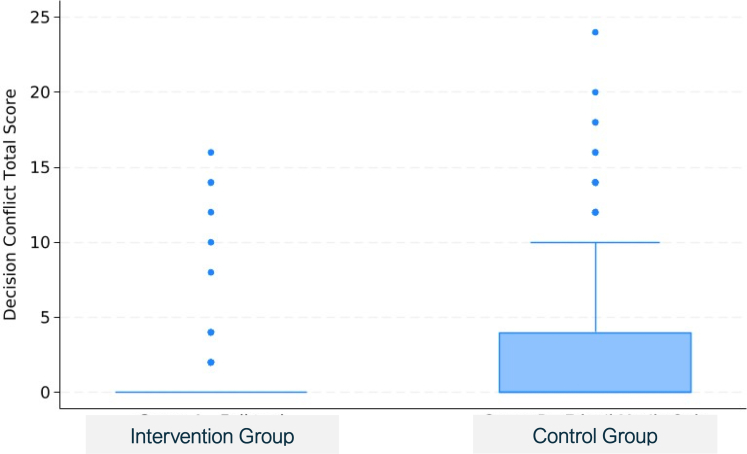
Decision conflict scale total scores by arm and box-and-whisker plots. For these right-skewed data, the lower boundary of the box is the median, whereas the upper boundary is the 75th percentile. The points are outliers more than 1.5 times the interquartile range above the 75th percentile.

**Fig. 6:**
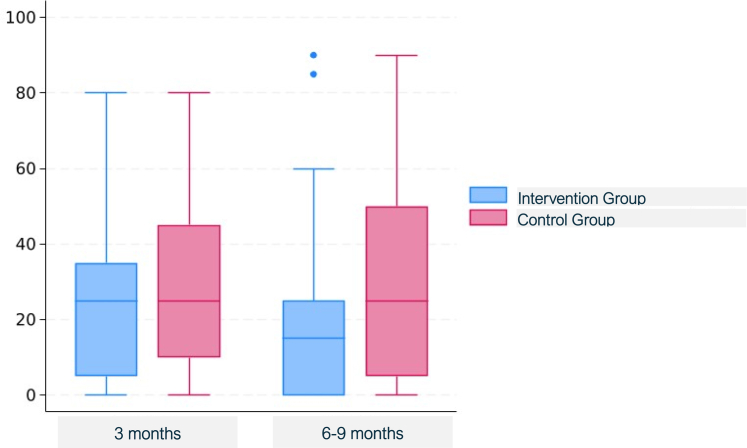
Decision regret scores, by arm, at 3-month (left) and 6–9 months (right) follow-up, box-and-whisker plots. The lower and upper bounds of the boxes are the 25th and 75th percentile, and the horizontal mid-line is the median. The points are outliers more than 1.5 times the interquartile range above the 75th percentile.

**Fig. 7:**
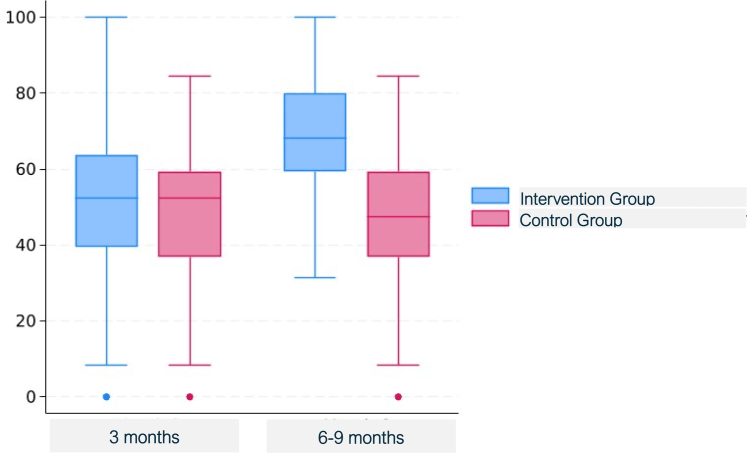
Knee injury and osteoarthritis outcome score joint replacement (KOOS JR), by arm, at 3-month (left) and 6–9 months (right) follow-up, box-and-whisker plots. The lower and upper bounds of the boxes are the 25th and 75th percentile, and the horizontal mid-line is the median. The points are outliers more than 1.5 times the interquartile range below the 25th percentile.

Patients in the intervention groups also had lower decision conflict scores (1.0 [3.1] versus 3.3 [5.8], *P* = 0.0029) (Fig. 5), lower decision regret scores at 6–9 months (18.2 [19.5] versus 27.2 [24.2] (Fig. 6), *P* = 0.0051), better physical function (KOOS JR) at 6–9 months (69.5 [17.3] versus 47 [18.4], *P* < 0.0001) (Fig. 7), and greater treatment concordance (91.1% versus 76.0%, *P* = 0.0043) compared with the control group; confidence intervals for treatment contrasts in Table 2.

In the intervention group, levels of SDM, physical function at 3 months, patient and clinician satisfaction, patient-clinician consultation duration, and TKA rates were higher than for the control group but were not statistically significantly different. Specifically the mean [SD] CollaboRATE score was 25.6 [3.9] versus 25 [3.6], *P* = 0.12; the 3-month KOOS JR interval score was 51.4 [20.1] versus 48.3 [17.4], *P* = 0.29; the NRS-P patient satisfaction score was 9.7 [1.2] versus 9.5 [1.1], *P* = 0.20; and the mean NRS-C clinician satisfaction score 9.2 [1.1] versus 9.1 [1.3], *P* = 0.36 for the intervention and control groups, respectively. Decision regret scores at 3 months were lower in the intervention group than in the control group (22.4 [20] versus 25.9 [20.4], *P* = 0.29). The mean patient-clinician consultation duration was 24.0 [10.7] minutes in the intervention group versus 22.5 [10.6] minutes in the control group (*P* = 0.36). In the intervention group, the TKA rate was 24.8% compared with 15.0% in the control group *P* = 0.11. All five of the *P*-values < 0.05 were still significant after multiple comparisons adjustment according to the Hochberg (1988) approach.

## Discussion

Despite their availability, widespread uptake of patient generated health data within solutions to augment SDM are lacking in the management of knee OA, a common and preference-sensitive condition associated with discretionary interventions such as TKA. The findings of this study highlight a strong case for adopting AI-DAs that combine patient-specific predictive analytics with patient education and preference assessment at the point of care to provide a personalized SDM experience. The full AI-DA used in this study demonstrated improved decision quality, medium- to longer-term physical function, and treatment concordance, while lowering levels of decision conflict and decision regret, compared with digital patient education only.

We previously conducted a smaller-scale RCT involving this AI-DA and found it to improve decision quality and knee-specific health during early follow-up. The current study involves a larger patient cohort and provides a more comprehensive, longer-term evaluation of knee-specific health and aspects related to effective decision making, including decision conflict and decision regret, in addition to decision quality. The current study also assessed clinician experience with the SDM interaction in addition to patient satisfaction, a variable often absent in other studies involving DAs.

While studies have evaluated DAs in patients with knee OA contemplating TKA, few incorporate a comprehensive assessment of impact on health outcomes and a range of decision-making characteristics both in the near-term and over a longer timeframe. A systematic review of RCTs evaluating proprietary DAs for knee OA underlined a strong focus on assessing decision quality (primarily knowledge transfer about treatment options using instruments such as the K-DQI) and less on extent of SDM, choice awareness, and deliberation (assessed using CollaboRATE, decision regret, and decision conflict scales), or health related outcomes (assessed using PROMs).^10^^,^^12^

The AI-DA in this study demonstrated higher levels of decision quality as well as less decision regret and decision conflict 6–9 months post-initial consultation compared with usual care. Our findings for K-DQI, our primary outcome, were particularly strong. To score 80% or above in the survey, participants would have to answer “some” or “a lot” on average to all 5 questions. Considering this threshold a clinically meaningful marker of high decision quality, 79.2% of patients experiencing the AI-DA scored 80% or above, compared to 60% receiving education alone (Table 2).

In contrast to these findings there was very little difference in level of SDM (measured by CollaboRATE). This instrument is described as a brief, ‘fast and frugal’ patient reported measure of SDM designed for use in routine clinical encounters. CollaboRATE assesses the level of effort in terms of ‘explanation’–helping patients understand their health issue, ‘preference elicitation’–listening to what matters most to patients about their health issue, and ‘preference integration’–including what matters most to patients in choosing the next steps in care.^22^ The K-DQI may offer a more direct assessment of the quality of the SDM process in the context of knee OA, treatment options, and the AI-DA experience (based on patient perceptions of being offered a choice, how much the pros and cons of different treatments including TKA and non-surgical alternatives were discussed, and whether clinicians engaged patients about their preferences and values to align these with treatment choice).^21^ Similarly, the DCS may also provide a more direct assessment of uncertainty versus effectiveness of decision making in relation to feeling the choice is informed, values-based, and likely to be implemented, alongside the level of satisfaction with the choice. Finally, the DRS may offer a more in-depth probe of deliberation and distress or remorse after a health care decision is made. Further research is required to better understand the fidelity of each tools to assess various decision-making characteristics in the context of data-informed, personalized DAs and their longitudinal impact on SDM.

Our prior RCT using the AI-DA showed improved levels of physical function at 3 months over the short-term.^18^ Although the current study also showed improved physical function at 3 months in the full AI-DA group, the difference with the control group was not statistically significant, possibly because substantial recovery in pain relief and function following TKA requires at least 3–6 months. The magnitude of change in level of functional outcomes and sustained improvement over 6–9 months from using AI-DAs during the initial consultation has not been demonstrated previously. Considering published thresholds for clinically meaningful benefit in KOOS JR (minimal clinically important difference, substantial clinical benefit, and patient acceptable symptom scale), a substantially greater proportion of patients experiencing the AI-DA achieved these thresholds compared to those receiving education only (Table 2).^19^^,^^27^ The sustained benefit in knee health through experiencing the AI-DA may be explained by the combination of components facilitating greater patient engagement, expectation management, and adherence to treatment plans—factors that are associated with improved physical function and reduced symptom intensity over the longer-term.^10^ In support of this effect, other studies have shown that patients who decline the use of DAs for hip or knee OA have greater symptom intensity and worse quality of life following surgical management for OA.^28^ Notably, we found that within the intervention group, both operated and non-operated groups had very similar outcomes, although we do not have statistical power to detect such differences. The enablement of clinician-patient dyads that make better, more appropriate treatment selections as to who may or may not benefit from surgery, and the AI-DA better preparing patients for next steps, irrespective of surgical or non-surgical treatment, should be studied further.

Patient and clinician satisfaction ratings were only marginally but not statistically significantly higher for patients assigned to the full AI-DA compared with patient education alone (Cohen’s *D* less than 0.2). Lack of difference may be partly due to the high ceiling effects experienced with the rating scale scores for satisfaction. The use of DAs incorporating high-quality digital content related to knee OA and its management has been shown by others to achieve high levels of patient satisfaction.^29^, ^30^, ^31^ Future studies should incorporate tools measuring patient satisfaction that limit the ceiling effect and more directly assess aspects of trust and confidence with the clinician during the SDM consultation.

Concerns over prolonged appointment durations, longer wait times for patients, and potential increases in non-operative management rates leading to lower procedural volumes have been expressed by orthopaedic surgeons in relation to DAs. While the mean appointment duration was longer with the full AI-DA than with patient education only, the difference was small and not statistically significant. Boland and colleagues demonstrated a difference in appointment times and outcomes following the use of DAs in academic versus community setting. Further testing of our AI-DA in different settings is important.

In relation to TKA rates, our study showed higher TKA rates with the use of the full AI-DA versus patient education alone, but again this difference was not statistically significantly different, albeit our study wasn’t powered to detect such differences. Surgical rates were generally low in this study and broadly consistent with the population experiencing a comprehensive whole person approach to lower extremity care at our academic medical center. In previous studies, the association between DA use and surgical rates has been highly variable. Some studies showed DAs to be associated with higher surgical rates,^32^ which may reflect a bias toward surgical procedures given that many of these tools don’t have an explicit wait-and-see option that reinforces evidence-based non-operative strategies such as structured physical therapy and guided self-management. Notably, patients declining the use of DAs altogether have also been shown to have a tendency to opt for surgery.^28^ In contrast, other studies showed that DA users tended to opt for more conservative treatments.^31^^,^^33^

Such variability underscores the general principle of not utilizing these tools as levers to dictate treatment choice in the care of musculoskeletal conditions. Rather, a DA (including the AI-DA in this study) should provide clear guidance on both evidence-based operative and non-operative strategies and be regarded as a data-informed guide for matching treatment choice with patient preferences, values, and needs. Our findings also showed significantly greater treatment concordance in the intervention group, suggesting that integrating the AI-DA within a lower extremity orthopaedic clinic can deliver care matched with patient choice. Further, patients in the intervention group were also more confident of their treatment preference than patients in the control group. The rates we found are not dissimilar to prior studies, which showed about 1 in 5 patients remain uncertain about their treatment preferences even after viewing a DA.

To date, evidence around outcome data-informed DAs has been mixed. Bansback et al., evaluated the effectiveness of an online DA combined with a surgeon report providing individualized potential outcomes of knee replacement surgery in a randomized controlled trial at a high-volume orthopaedic clinic.^34^ Provided during the pre-surgical assessment, these tools showed improved decision quality predominantly through increased knowledge scores. The same research team performed a further RCT using the combined tool but demonstrated equivocal findings compared to usual care in relation to level of decisional regret, patient satisfaction, health-related quality-of-life, and meeting patient expectations.^35^ Authors suggested the mixed outcomes being potentially due to patients and clinicians not having sufficient time to engage with tools within a busy clinical setting already providing high volume surgical care. These findings highlight an opportunity for integration and evaluation of personalized DAs earlier in the care pathway where patients may encounter greater opportunities and time for deliberating different treatment strategies including non-surgical options.

Other studies have also utilized AI in care for patients with knee OA. Harris and co-authors conducted a study involving 587 veterans undergoing TKA; despite utilizing a large and robust dataset to develop patient-specific predictive models and achieving a 92% completion rate of the KOOS at 1 year, the study was not able to demonstrate minimal clinically important differences in KOOS scores 1 year after TKA.^17^^,^^36^ Franklin and co-workers also conducted a large-scale implementation of AI incorporating pre-visit PROs and clinical risk factors to facilitate decision support for patients with hip and knee OA who used a web-based tool providing a patient-specific outcomes report.^37^ They reported, however, uncertainty around whether the report was reviewed by both parties, underscoring the importance of viewing the report ahead of an interactive discussion with the clinician. In our study, patients in the intervention group and their orthopaedic surgeon always reviewed the personalized report prior to the consultation. Others have also demonstrated the feasibility of utilizing ML to estimate post-operative PROM scores by using a combination of self-reported and clinical data for predicting achievement of a minimal clinically importance difference in PROM scores for physical function and symptom at 1 year following TKA.^38^, ^39^, ^40^

The findings in our study underscore a strong use-case for PROMs adoption and applying these measures to form A.I-generated digital twins with person-specific analytics that can augment decision making. PROM collection has generally increased in orthopaedics, largely due to quality reporting requirements by payers and advanced accreditation programs rather than capture and application of scores at the point-of-care. A.I can be leveraged to achieve this unmet need, especially considering its growing acceptance by users on the frontlines of care delivery. We envision the increased availability of patient generated health data to contribute to a comprehensive set of data inputs capable of generating digital twins that not only predict outcomes of surgical treatments but eventually computational models that forecast the impact of a full range of non-operative strategies (physical therapy, behavioral therapy, and lifestyle interventions), and combinations of these interventions.

The AI-DA in this study combines content delivery prior to the consultation (information on probabilities communicated in numbers), strategies to decrease cognitive demand (educational material with options presented side-by-side), implicit values clarification (person-specific predictions stimulating patients to independently weigh-up the desirability of surgical versus non-surgical care and imagine what the treatment experience might be like), explicit values clarification (rating scales to actively compare the relative importance of features of different treatment options), and strategies to facilitate discussion (the personalized report). Further studies are required to evaluate the relative impact of these important attributes of DAs to advance design, development, and evaluation prior to deployment.^41^

There are several limitations to this study. First, the RCT was performed at a single university academic health system providing comprehensive, whole person musculoskeletal specialty care, which is relatively uncommon compared to usual care and particularly centers performing high volume joint replacement surgery. Non-operative strategies tailored to the patient’s needs can avert or divert surgery in approximately 15–20% of patients eligible for TKA. This clinical setting may limit the generalizability of our findings until more centers overcome human (cultural shift from procedure and volume-focused care, toward condition-oriented, value-based care), clinical (e.g., development of non-operative services and personnel), and technical barriers (e.g., development of coding, payment, and prior-authorization arrangements) to expand their selection of non-operative strategies. While our current study involving the AI-DA supports many of the findings of our prior trial, external validation and applicability of our findings, especially at non-academic centers, and particularly those that are traditionally anchored in volume-focused care is needed. This requirement is especially important considering the lack of formal peer-reviewed publication on original development, testing, and validation of the AI/ML model, which is a common issue across such proprietary algorithms in the digital health care arena.

Second, the orthopaedic surgeons involved in the SDM consultation were not blinded to study group and thus may have engaged differently with the intervention group than with the control group. Accounting for this form of bias is challenging given that these clinicians are proponents of SDM and the use of PROMs was routine in their practice. Future studies could incorporate more formal coaching for clinicians, including a more structured discussion guide, especially for those less experienced in formal SDM. Notably, some DAs include a communication aid to steer clinician-patient interactions.

Third, a statistical concern is that 21 participants were excluded from the analysis post-randomization; however, the number was balanced across study arms, minimizing concerns about bias owing to this occurrence. Further, we did not control for multiple comparisons or hypothesis tests. Even had we done so, it is unlikely to have changed the results given that our findings were either strongly significant (i.e., small *P*-values) or yielded *P*-values far from the nominal 0.05 threshold.

Fourth, the process of SDM should ideally allow for sufficient time for patients to read, process, and question information within the DA. Our study protocol was designed to fit within the normal clinical workflow, which meant that patients received the full AI-DA (or patient education alone) before their discussion with the orthopaedic surgeon, all within the same clinic visit. Alternatively, patients could receive the DA or its components earlier (e.g., remotely via a patient portal) thereby offering more time to examine the content prior to their appointment.

Fifth, while we prioritized the focus on the SDM process and treatment concordance as measures of decision quality, underscoring our rationale for excluding the evaluation of knowledge scoring, future studies should provide a more in-depth evaluation of assessment, planning, implementation, and assessment of patient education content during patient-centric decision support for knee OA care.

Finally, a concern with any AI-enabled clinical decision support solution is algorithmic bias. The AI/ML algorithm does not explicitly adjust for variations such as type of clinical setting, surgical procedural differences, or level of surgeon training. Further, the model was also trained without race or ethnicity as inputs. In the US, racial and ethnic minorities, specifically those self-identifying as black or Hispanic are prone to disparities related to access to musculoskeletal care and surgical treatment selection, and shown to have lower levels of trust, confidence, perceptions of benefit, and preference for joint replacement surgery compared to white patients.^42^ The large population level dataset encompassing several US geographies used to train the algorithm supports generalizability and potentially mitigates the level of bias. Further, the 2 groups in this study were well balanced by race and ethnicity. However, future work is needed to more closely examine the impact of clinical variations and individual preferences related to race, ethnicity, and social factors in the context of AI-DAs and person-specific predictive models.

In conclusion, an escalation in TKA rates and dissatisfaction among a sizeable group of patients following surgery, triggers opportunities for utilizing DAs that incorporate individualized benefits and risks alongside patient preferences to facilitate treatment selection. It is incumbent upon clinicians who treat knee OA to utilize all available data to identify those most likely to benefit treatment. Our study provides foundational evidence for AI-DAs featuring patient education, preference assessment, and patient-specific predictive analytics incorporating PROMs. The AI-DA improves aspects of decision making, levels of knee specific health, and treatment concordance compared with usual care. The utility of such tools will depend on their integration into clinical and technical workflows. Ongoing assessment will be important as the AI-DAs–specifically the computational model—continues to improve through learning from local data (i.e., from patients experiencing the tool) being fed back into the model. The development of AI/ML models that utilize clinical data (including longitudinal PROMs) related non-operative treatments will further enhance the impact of the tool through person-specific information for all treatment strategies and not TKA alone. Systematic incorporation of patient preference data accounting for racial, ethnic, and sociodemographic variations may also enable the model to assist in reasoning during decision-making, mirror individual behavior, and map behavioral patterns accounting for different population phenotypes. The next generation of AI-DAs will require rigorous cost effectiveness analysis and external validation including multi-center trials to support universal adoption of digital twins for patient centric decision support at the point of specialty care.

## Contributors

Jayakumar, Bozic, and Tsevat had full access to all the data in the study and takes responsibility for the integrity of the data and the accuracy of the data analysis.

Conceptualization: Jayakumar, Uhler, Andrawis, Koenig, Rathouz, Tsevat, Bozic.

Data curation: Jayakumar, Uhler, Lin, Trutner.

Investigation: Jayakumar, Uhler, Lin, Trutner.

Methodology: Uhler, Tsevat, Bozic, Rathouz, Jayakumar.

Writing—original draft: Jayakumar, Tsevat.

Writing—review & editing: Jayakumar, Lin, Trutner, Uhler, Andrawis, Koenig, Rathouz, Tsevat, Bozic.

Data access and verification: Jayakumar, Tsevat, Rathouz.

Formal analysis: Jayakumar, Lin, Trutner, Jayakumar, Rathouz.

Visualization: Jayakumar, Tsevat, Rathouz.

Funding acquisition: Uhler, Andrawis, Tsevat, Bozic.

Project administration: Jayakumar, Lin, Trutner, Uhler, Andrawis.

## Data sharing statement

Data will be made available to researchers whose proposed use of this information has been approved. The data must only be used for the purposes specified in the data request. Data will be provided as deidentified participant data accompanied by supporting documentation including a data dictionary and statistical analysis code. Data will be transferred only after approval of a proposal and completion of a signed data access agreement. Access to these data and accompanying documents will granted following publication by submitting requests to: lauren.uhler@austin.utexas.edu under the supervision of Drs. Jayakumar, Koenig, and Bozic.

## Declaration of interests

**PJ:** Personal fees from SeniorCare Labs, CODE Technologies; Stock options from ORA Medical, Full Circle, Protera Health, Camino Robotics. Employment with Optum Insight, United Health Group.

Dr. Bozic and Dr. Jayakumar are co-developers of the Joint Insights tool with their University affiliation; they have no personal financial interest in the tool.

**EL:** Nothing to disclose.

**ZT:** Nothing to disclose.

**LMU**: Nothing to disclose.

**JA:** Personal fees from Sylke Inc.

**KMK:** Personal fees from National Peer Review Corporation, Academic Orthopaedic Consortium Corporation.

**PR:** Through employment at UT Austin, PR receives salary support from NIH, foundations, and a State agency. He served on a DSMB for Sunovion Pharmaceuticals and will serve on a DSMB for Syneos.

**JT**: Royalties from Wolters Kluwer.

**KJB**: Personal fees from the CMS and Purchaser Business Group on Health; stock options from Carrum Health; leadership role with the American Academy of Orthopaedic Surgeons; royalty agreements with Wolters Kluwer and Slack Incorporated. Dr. Bozic and Dr. Jayakumar are co-developers of the Joint Insights tool; they have no personal financial interest in the tool.

The University of Texas at Austin has a royalty agreement with OM1, Inc.

## Appendix A Supplementary data

Supplemental File. Missing_Value_Table_v1

Supplemental File. Outcome_Measures_Scoring_Methodology_v1

JointInsights_OpProtocol v1.6
