# Supplementary material for: Shared decision making using digital twins in knee osteoarthritis care: a randomized clinical trial of an AI-enabled decision aid versus education alone on decision quality, physical function, and user experience
Source: eClinicalMedicine. 2025 Oct 4;89:103545. doi: 10.1016/j.eclinm.2025.103545 (PMC12528923; doi:10.1016/j.eclinm.2025.103545)
Supplement: Supplemental File. Missing_Value_Table_v1 [file mmc1.docx]

**Supplemental File. Missing Value Count for Outcome Variables**

| Outcome Variable | Complete observations | Missing observations | Unique Values | Minimum | Maximum |
| --- | --- | --- | --- | --- | --- |
| DRS at 3-month | 166 | 35 | 16 | 0 | 80 |
| DRS at 6-month | 167 | 34 | 18 | 0 | 90 |
| KOOS JR Score 3-month | 188 | 13 | 28 | 0 | 100 |
| KOOS JR Score 6-month | 193 | 8 | 29 | 0 | 100 |
