# Supplementary material for: Shared decision making using digital twins in knee osteoarthritis care: a randomized clinical trial of an AI-enabled decision aid versus education alone on decision quality, physical function, and user experience
Source: eClinicalMedicine. 2025 Oct 4;89:103545. doi: 10.1016/j.eclinm.2025.103545 (PMC12528923; doi:10.1016/j.eclinm.2025.103545)
Supplement: Supplemental File. Outcome_Measures_Scoring_Methodology_v1 [file mmc2.docx]

**Outcome Scoring Methodology**

The Knee Decision Quality Instrument (K-DQI) (21) is survey containing three sets of items resulting in three separate scores: a total knowledge score, a concordance score, and an SDM Decision Process Score. For the purposes of this study, we prioritized the SDM Decision Process Score and concordance score as direct measures of decision quality. Our complete rationale is provided in the manuscript.

The SDM Decision Process Score includes in **“Section 3. Talking with Health Care Providers”** (see below)**,** patients being asked about whether they were offered a choice, how much the pros and cons of different treatments were discussed, and whether the health care provider asked about their preferences (see below).

We used both the original and the modified scoring method. In the original scoring method, participants received 1 point for a response of “yes” or “a lot / some”. The total points are summed and then divided by the total number of items to result in scores from 0-100%, with higher scores indicating a more shared decision-making process.

In the modified scoring method, participants received 1 point for a response of “yes” or “a lot”, 0.5 point for “some”, and 0 point for all other responses. The total points are summed and result in total scores from 0-5, with higher scores indicating more shared decision making. The scoring for this scale (detailed in the accompanying table below) was revised in 2020 to provide partial credit for responses of ‘some’ = 0.5.

**Section 3. Talking with Health Care Providers**

**Please answer these questions about what happened when you talked with health care providers including** **doctors, nurses and other health care professionals about knee replacement surgery and other non-surgical** **treatments, such as exercise or medicine, for knee osteoarthritis.**

**3.1**. Did any of your health care providers talk about knee replacement surgery as an option for you?

ο Yes

ο No

**3.2.** How much did you and your health care providers talk about the reasons to have knee replacement

surgery?

ο A lot

ο Some

ο A little

ο Not at all

**3.3.** How much did you and your health care providers talk about the reasons **not** to have knee replacement

surgery?

ο A lot

ο Some

ο A little

ο Not at all

**3.4.** Did any of your health care providers talk about non-surgical treatments as something that you should

seriously consider?

ο Yes

ο No

**3.5.** Did any of your health care providers ask you whether you wanted to have knee replacement surgery or

not?

ο Yes

ο No

**Scoring Table**

*
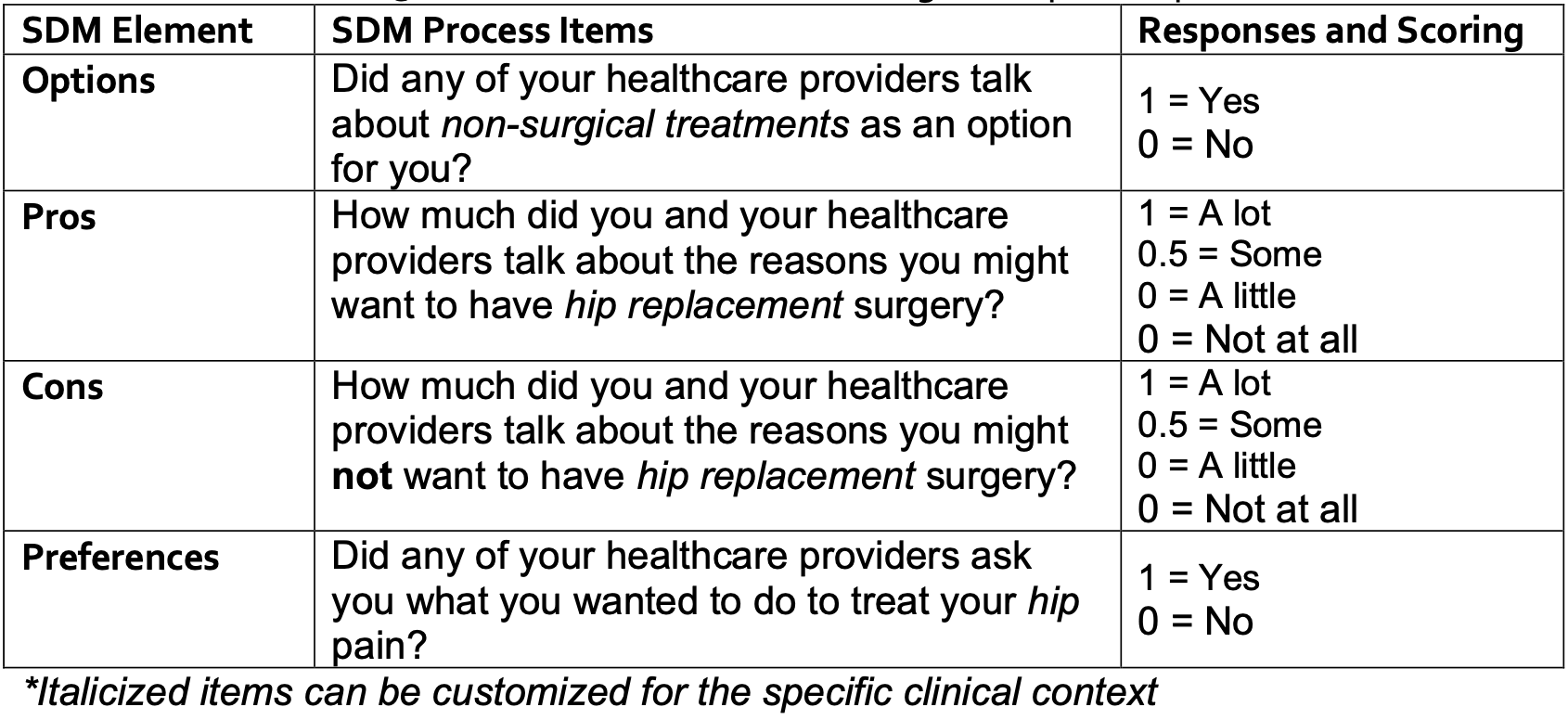
*

The concordance score was calculated using the direct, simple match method, where the patients’ preferred treatment is assessed with the single item below and then compared with the treatment received to determine whether they matched. Patients who are unsure are not considered to have a treatment that matches. A summary score (0-100%) can be generated to indicate the percentage of patients who received treatment that matched their stated preference.

**Section 1. What Matters Most to You?**

**1.6.** Which treatment do you want to do to treat your knee osteoarthritis?

ο Knee replacement surgery

ο Non-surgical treatment options

ο I am not sure

**Additional References:**

**K-DQI Worksheet:** <https://mghdecisionsciences.org/wp-content/uploads/2024/02/DQI_Knee_Osteoarthritis_v2.0_sv_2024.pdf>

**K-DQI User Guide:** <https://mghdecisionsciences.org/wp-content/uploads/2024/02/DQI_Hip_Knee_Osteoarthritis_v2.0_User-_Guide_2024.pdf>

The Decision Conflict Scale (DCS) (23) is a 10-item survey, with each response value summed, divided by the total item number and multiplied by 25. The score ranges from 0 to 100, where 0 is no decisional conflict and 100 is the greatest decisional conflict.

The Decision Regret Scale (DRS) (24) measures distress or remorse after making a healthcare decision. The answer values are summed and converted to a 0–100 scale, where a higher score indicates more regret. BMI, body mass index; ED, emergency department; TKR, total knee replacement.

The CollaboRATE (22) is a three-item, 10-point anchor scale measuring the level of shared decision-making in a clinical encounter. It yields a continuous score with a possible range from 0 to 100, where higher scores represent a greater degree of shared decision-making.

The Knee injury and Osteoarthritis Outcome Score for Joint Replacement (KOOS JR) (19) is a seven-item patient-reported outcome measure of knee joint-related stiffness, pain and function; interval scores range from 0 to 100, with 0 representing poorest knee health and 100 best knee health.

The Patient-Reported Outcomes Measurement Information System (PROMIS) Global-10 (20) is a 10-item measure assessing health-related quality of life with items about overall physical and mental health including social connections and physical capabilities. The survey is scored using two sub scores, one for physical health and one for mental health, where specific items are used for a raw score and then converted to a T-score. Population norm T-scores are 50 on each sub score; higher scores reflect better functioning.

Source: Adapted from Lin E, Uhler LM, Finley EP, Jayakumar P, Rathouz PJ, Bozic KJ, Tsevat J. Incorporating patient-reported outcomes into shared decision-making in the management of patients with osteoarthritis of the knee: a hybrid effectiveness-implementation study protocol. BMJ Open. 2022 Feb 21;12(2):e055933. doi: 10.1136/bmjopen-2021-055933. PMID: 35190439; PMCID: PMC8860037.
