# Supplementary material for: Shared decision making using digital twins in knee osteoarthritis care: a randomized clinical trial of an AI-enabled decision aid versus education alone on decision quality, physical function, and user experience
Source: eClinicalMedicine. 2025 Oct 4;89:103545. doi: 10.1016/j.eclinm.2025.103545 (PMC12528923; doi:10.1016/j.eclinm.2025.103545)
Supplement: JointInsights_OpProtocol v1.6 [file mmc3.docx]

**Impact of an Artificial Intelligence-Enabled Decision Aid on Decision Quality, Shared Decision Making, Patient Experience and Functional Outcomes in the Management of Knee Osteoarthritis: A Randomized Clinical Trial**

**PROTOCOL VERSION:** 1.3

**VERSION DATE:** 11.11.21

**Administrative Information**

***Trial Information***

Trial Number: NCT04805554

Trial Registry: ClinicalTrials.gov

Date of Registration: March 18, 2021

Contact: Lauren Uhler, MPH (512) 495-5090

Funding: AHRQ Grant R21HS027037

Coordinating Center: Dell Medical School at the University of Texas Austin

Ethics Review: Initial approval by UT IRB on February 21, 2020 (#2018110042, modifications approved later)

Study Type: Interventional

Study Design: Randomized parallel assignment open label (unblinded) study

Sample Size: 200

Date of First Enrollment: February 22, 2021

Recruitment Status: Enrolling

Expected Completion: June 2022

Sponsor: The funder (AHRQ) has no role in study design, collection, management, analysis, or data interpretation. The funder has no role in the writing of the report or the decision to submit the report for publication.

Principal Investigators: Prakash Jayakumar; Joel Tsevat; Kevin J. Bozic

Grant Holders: Kevin J. Bozic and Joel Tsevat

Protocol: Dell Medical School: Kevin J. Bozic (KJB), Lauren Uhler (LU), Prakash Jayakumar (PJ), Eugenia Lin (EL), Zoe Trutner (ZT), Karl M. Koenig (KMK) Paul Rathouz (PR). UT Health Science Center San Antonio: Joel Tsevat (JT)

Roles: Clinical trial design / conception: PJ, JT, KJB, PR, KMK; Study initiation: PJ, JT, KJB, LU; Study implementation / operations: EL, ZT, KMK, KJB; Statistical analysis: PR; Study protocol and refinement: All authors; Synthesis of data analysis: All authors; Approval of final manuscript: All authors.

Data Management: PJ, Lauren Uhler

Recruitment: EL, ZT

**Introduction**

***Background***

In the era of patient-centered care, there is a growing interest in educating and engaging patients in the clinical decision-making process. This sets patients and providers up for making the right treatment choices for a medical condition (Sepucha 2017)(duLong 2016)(Bozic 2013). Actively working through treatment options and using decision aids as incorporated into the clinical work flow is shown to generate positive attitudes amongst patients and providers during consultations (Williams 2017)(Mangla 2018)(duLong 2016).

The concept of shared decision-making utilizing decision aids has been advocated as a more patient-centered approach to orthopaedic surgical care (Mangla 2018)(duLong 2016)(Stacey 2014). Studies have demonstrated the positive impact of decision aids on patient knowledge, efficiency and quality of decision-making, treatment choice, and patient and surgeon experience in treatment of hip and knee OA (Bozic 2013,Stacey 2014). The use of decision aids to generate dialogue and involve patients in the decision-making process may also enable more comprehensive discussions around all available treatment options. In this way, utilization of care may be increased in the management of joint pain secondary to osteoarthritis (Hofstede 2016). In association, the use of decision aids in routine orthopaedic care has shown reduced surgical rates, due perhaps to more informed discussions and decision-making (Sepucha 2017).

Decision aids may also contend with the influence of socioeconomic factors on treatment choices (Youm 2015). Those patients with less education and lower incomes are shown to be 1) less knowledgeable about their medical conditions and treatment options and 2) less able to arrive at a decision following consultations for joint pain related to osteoarthritis (Youm 2015). One study involving shared decision-making in the treatment of hip and knee osteoarthritis demonstrated the influence of the doctor-patient relationship on decision-conflict with a need for more information and clarity around the risks and benefits of surgery (duLong 2016).

This study evaluates a technology-enabled decision aid (OM1, Boston, MA, USA) that provides evidence-based education (i.e. on understanding the condition, treatment options and comparisons, patient values and question & answer quiz), preference assessment, and personalized, quantifiable benefit-risk ratio alongside the level of potential benefit in relation to joint stiffness, joint pain and quality of life. The study will be performed in the setting of an integrated care system providing patient-centered care for hip and knee OA over the full cycle of care.

The impact of a technology-enabled decision aid incorporating predictive analytics with machine learning capabilities on decision quality, activation, experience & functional outcomes of patients in this setting is relatively unknown. As a comparator or control group, this study will utilize identical evidence-based educational materials but will exclude the personalized risk ratio report and preference assessment.

***Objectives***

Assessing impact of a technology-enabled full decision aid (patient education, preference assessment, patient-specific predictive analytics) on decision quality, functional outcomes, concordance, shared decision making, patient/ provider satisfaction with consultation discussion, decisional conflict, and decision regret, consultation time, rates of surgical treatment compared to a control arm (patient education only).

***Trial Design***

This is a two-armed parallel group randomized controlled trial with an allocation ratio of 1:1 using block randomization that is testing the impact of a full technology-enabled decision aid (patient education, preference assessment, person-specific predictive analytics) compared to educational material only.

**Methods**

***Setting***

Subjects will be identified and recruited through the Lower Extremity Musculoskeletal Institute (MSKI) Clinic, an integrated practice unit at the University of Texas at Austin / Dell Medical School which is an urban, academic, tertiary care center. The subjects are identified as new patients presenting with knee pain with a presumptive diagnosis of OA and a scheduled appointment with KJB or KMK.

***Eligibility Criteria***

Inclusion Criteria (to be checked during huddle/ after check-in and x-ray/ at consent):

1. All new patients with a presumptive diagnosis of knee OA
2. Aged between 45 and 89
3. K-L grade of 3 or 4 (moderate to severe)
4. KOOS JR scores 0-85
5. Able to provide informed consent

Exclusion Criteria (to be checked during huddle/ after x-ray and check-in/ at consent):

1. Prior total knee replacement
2. Prior consultation with an orthopaedic surgeon for TKR
3. Prior experience with Joint Insights
4. Patients undergoing consideration for revision joint replacement
5. Patients seeking care for a trauma condition, or psoriatic/rheumatoid arthritis
6. Non-English or Non-Spanish speakers
7. Patients with BMI below 20 or above 46

***Interventions***

Intervention Arm: “Full Tool”

- Prior to consultation with a provider patients experience:
  - Demographic survey
  - Patient reported outcomes surveys:
    - PROMIS-Global 10
    - KOOS JR
  - OM1 Joint Insights educational module
  - OM1 Joint Insights preferences module
  - OM1 Joint Insights score report
- During consultation with a provider patients experience:
  - Discussion about the results of the decision aid
- Immediately after consultation with a provider patients experience:
  - Decision quality and satisfaction surveys:
    - DQI
    - CollaboRATE
    - DCS
    - Satisfaction score
- 3 and 6-9 months after consultation with a provider patients experience:
  - Decision quality surveys
    - DRS
  - Patient-reported outcomes surveys
    - KOOS JR
    - PROMIS-Global 10
  - *Patients may request to leave the study at any point without repercussion*
  - *Patients are contacted via phone, email, text, and/or Athena MyChart*
  - *Patients may participate in any concomitant care or interventions without limitation*
- 6-9 months after consultation with the provider and completion of all follow-up surveys:
  - Patients receive via mail or email a $25 gift card

Control Arm: “Education Only”

- Prior to consultation with a provider, patients experience:
  - OM1 Joint Insights educational module
- During consultation with a provider patients experience:
  - Standard care *without* discussion of the OM1 Joint Insights modules
- Immediately after consultation with a provider patients experience:
  - Decision quality and satisfaction surveys
- 3 and 6-9 months after consultation with a provider patients experience:
  - Decision quality surveys
  - Patient-reported outcomes surveys
  - *Patients may request to leave the study at any point without repercussion*
  - *Patients are contacted via phone, email, text, and/or Athena MyChart*
  - *Patients may participate in any concomitant care or interventions without limitation*

***Allocation***

Sequence Generation: REDCap algorithm (see details below)

Concealment: Sequence concealed by REDCap algorithm

Implementation: Research Assistant (see protocol below for Randomization)

Blinding: Unblinded/ no masking

For the randomization, we will stratify by variables known or suspected to be predictive of decision quality and/or treatment choice by using stratified random block randomization. Participants will be stratified by 3 factors: ethnicity (Hispanic/non-Hispanic), insurance (public safety net insurance/other insurance) and attending surgeon. Block randomization will occur within each stratum with random block sizes of 4 and 6, and an equal number of patients assigned to the intervention and control arms within each block. These steps are carried out automatically in the REDCap study database. This strategy ensures that participants are equally distributed to treatment and control through time, therefore controlling for any time varying effects that may occur. The Dell Medical School Biomedical Data Science Hub will oversee the randomization.

***Outcomes***

This study will assess the impact of a technology-enabled decision aid on:

| **Outcome** | **Measure** |
| --- | --- |
| Decision quality | Knee Decision Quality Index Q3.1-3.5 |
| Concordance | Knee DQI Q1.6 & whether patient had TKA within 9 months (Athena) |
| Shared decision making | CollaboRATE survey |
| Patient/provider satisfaction with discussion | Satisfaction question |
| Consultation time | Duration of surgeon/patient consultation in minutes as measured by research assistant |
| Overall health | PROMIS Global 10 |
| Change in limitations | KOOS JR |
| Treatment selected | Treatment choice/arthroplasty rate |
| Decisional conflict | Decisional Conflict Scale 10 (low literacy) |
| Decision regret | Decision Regret Scale |

***Sample Size***

The aim is to enroll a total of 200 subjects with randomization to either the intervention arm or the control arm of the study (100 controls and 100 interventions). The study is powered to account for 10% loss to follow-up, with a target sample size of 180 patients. We calculated the sample size for the RCT by treating the Decision Process score of the DQI as continuous. We aimed to detect a treatment effect size (i.e., Cohen’s D) as small as 0.5 (consistent with preliminary data from the first 26 subjects we have studied) with a type I error rate of 0.05 and power of 0.90, assuming equal sample size in intervention and control groups. Given our 8 randomization strata, we estimate a needed sample size of 180 participants, or 90 for each group. With an estimated loss-to-follow-up rate of 10%. Our target enrollment for the RCT is 200 participants, or 100 for each arm.

***Timeline***

Recruitment Start: February 2021

Projected Recruitment End: August 2022

Projected Follow-Up End: November 2022 (follow-up at 3- and 6-9 month time points)

***Recruitment & Follow-Up Flowsheet***
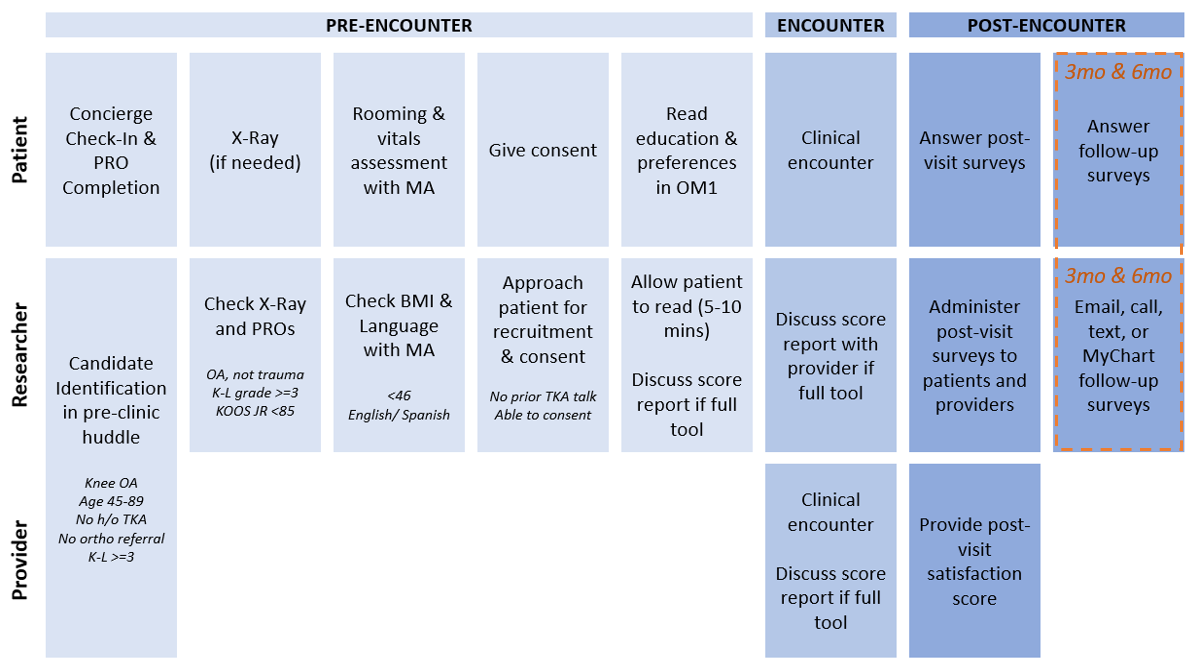


***Recruitment methods: Eligibility Screening***

Patients will be identified as suitable candidates for the study by members of the research team during the pre-clinic meeting (huddle). Initial decisions will be made based on appointment type, chief complaint, age, availability of radiographs, and availability of a recent BMI.

Once the patient has completed any required imaging, entered the clinic room or private consultation space, and been checked in by the Medical Assistant (including measurement of height and weight), the researcher will confirm that the patient meets all inclusion criteria and none of the exclusion criteria. At this time, the researcher should record the patient’s height and weight to facilitate enrollment in the OM1 tool. Additionally, the researcher should prepare translation services if necessary. The researcher will then approach the patient and invite him or her to participate in the study.

- Eligibility Screening Documentation
  - All new patients with a presumptive diagnosis of knee OA aged 45-89 will be entered into the Excel worksheet **“Joint Insights Eligibility Screen”** (in Box)
  - Documentation will include:
    - Enrollment Status:
      - Consented (Y/N)
      - Screen Fail (Y/N)
      - Reason for screening failure:
        - Not OA
        - Prior TKA
        - Prior discussion with an orthopaedic surgeon about TKA
        - Previously seen/used Joint Insights
        - Considering revision joint replacement
        - Seeking care for trauma condition
        - Non-English or Non-Spanish speakers
        - K-L Joint space narrowing grade 1 or 2
        - KOOS JR score >85
        - BMI below 20 or above 46
        - Age under 45 or over 89
        - Unable to give informed consent
        - Other
      - Declined (Y/N)
      - Reason for declining:
        - Not enough time
        - Privacy concerns
        - Not interested
        - No reason given
        - Other (record reason)

***Recruitment methods: Informed Consent***

If the patient qualifies for the study, the researcher will obtain informed consent by adhering to the following script as much as possible:

*“Hi, my name is ______. I am part of the research team working with Dr. Bozic / Dr. Koenig, your physician today, on a study involving software that aims to help patients and their doctors make treatment decisions related to knee arthritis. This tool provides educational materials on your condition—the reason for your knee pain—and uses your age, sex, BMI and questions about health you already answered on the iPads to make personalized calculations of risks and benefits related to treatment of your knee pain.*

*The purpose of this study is to learn more about the impact of the software on aspects of decision making and assess your satisfaction with the decision making process. This will be done using questionnaires at the end of the visit. We will also email, call, text, or message you through the Athena portal at 3 months and 6-9 months from now to ask a few more questions about your treatment decision and see how your knee pain is doing. If you agree to participate, we will let you know whether you were randomly selected to a group receiving educational materials and a risk-benefit report produced by the software or to a group receiving educational materials only.*

*This is a fair way of studying an intervention, in this case the software, to learn about its impact. Regardless of what group you are in, after consultation you will be asked to complete a set of questionnaires that should take less than 10 minutes. Questions will be about the treatment discussion with your doctor and the decision-making process.*

*The tool* ***does not*** *replace the usual care you receive at our clinic so you will still see all the health care professionals you would usually meet and be able to discuss all the things you want to discuss with each member of our team.*

*Your participation is completely voluntary and does not impact whether you will receive care. No penalty will be applied should you decide not to participate. Do you have any questions? Do you agree to take part in the study regardless of the group you may be assigned? Would you like more time to think about it?”*

- If the patient chooses to participate
  - *“Thank you so much for your agreement to participate. I will now momentarily randomize you into either Group A or Group B. Regardless of which group you are assigned, your continued participation is very important for our study outcomes.”*
  - The researcher will then move on to enrollment and randomization (see below)
- If the patient declines to participate…
  - *“That’s okay. Could you kindly tell me your reason why? This is useful for feedback and enables us to improve our studies in future.”*
  - The researcher will then leave the room and inform the providers the patient is ready to be seen
- Regardless…
  - At any time, potential subjects can request additional time to think about the study or decline participation. All questions the patient may have will aim to be answered to the best of their ability by the research assistant, or the assistant may escalate questions to the project leaders or PI.

***Recruitment methods: Randomization***

The researcher will then enroll the patient and perform randomization using the REDCap online platform. Patients will be informed if they are in the intervention group (“Full Tool”) or control group (“Edu Only”). Researchers will do their best to follow standard scripts for each step of the recruitment as below.

- Prior to entering the room, the researcher should:
  - Open and log into REDCap
  - Open and log into OM1
- REDCap randomization procedure on clinic iPads, in room with patient:
  - From the REDCap homepage, enter the “Joint Insights” project
  - Go to Add/Edit Records on the left hand side of REDCap, then “Add new record”
  - Complete the “Informed Consent and HIPAA” record, including a signature
  - Complete the **first three items** on the “Enroller: Initial Visit Details” record
    - Site & clinic-associated surgeon (Bozic vs Koenig)
    - Is the patient Hispanic? (Y vs N)
    - What kind of insurance? (MAP/Medicaid vs Other)
  - Click the “Randomization” button, which will show either:
    - “Group A – Full tool”
    - “Group B – Educational materials only”


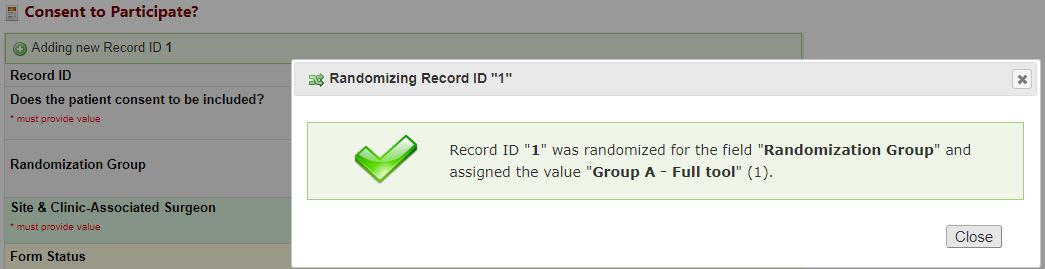


- - Inform the patient as to the group into which they have been randomized
    - *FULL TOOL: “You have been selected to receive the educational materials and the software. Let me briefly talk you through the process. Please kindly read the education material on this iPad at your own pace, answer the questions at the end, and use the sliders to tell us about your preferences and experiences. You will then receive a report customized to you which can guide discussion with your provider. Please feel free to ask any questions during this consultation. Thanks in advance for completing some survey questions on how it all went at the end of the visit, please be sure to wait for me to return before leaving.”*
    - *EDUCATION ONLY: “You have been selected to receive the educational materials. Let me briefly talk you through the process. Please kindly read the education material on this iPad at your own pace. You will then have a chance to discuss your problem and treatment options with your provider. Please feel free to ask any questions during this consultation. Thanks in advance for completing some survey questions on how it all went at the end of the visit, please be sure to wait for me to return before leaving.”*

***Recruitment Methods: OM1 Enrollment & Delivery***

After Randomization, leave REDCap and open instead the online OM1 platform in a new browser tab. Follow the procedure below to enroll the patient and open the education:

- Click “Add Patient” in the top menu bar
- Complete only the fields in the REGISTRATION and CONTACT PREFERNCES sections marked with an Asterix *
  - First name
  - Last name
  - Date of birth
  - Sex at birth
  - Language Preference
- Click “Continue”
- Complete all fields in the CARE TEAM section
  - Organization (UT Health Austin)
  - Physicians (Bozic vs Koenig)
  - Musculoskeletal (L vs R knee)
- Click “Save”
- Complete all fields with an Asterix * in the [RIGHT/LEFT] KNEE section
  - Type
    - “TKA Consultation” if patient is in Group A / Full tool
    - “TKA Education” if patient is in Group B / Educational materials only
  - Event date (today)
  - Facility (UT Health Austin)
  - Physician (Bozic vs Koenig)
  - *If patient is randomized to the Full Tool, the below fields will also appear, which the researcher will then complete using the medial chart or by speaking directly with the patient:*
    - Height (manually enter from chart)
    - Weight (manually enter from chart)
    - Tobacco use (select one)
    - Comorbidities (select all that apply)
    - #ED visits in the last year
    - #Hospitalizations in the last year
- Click “Save”
- The following screen will automatically appear after a few moments:


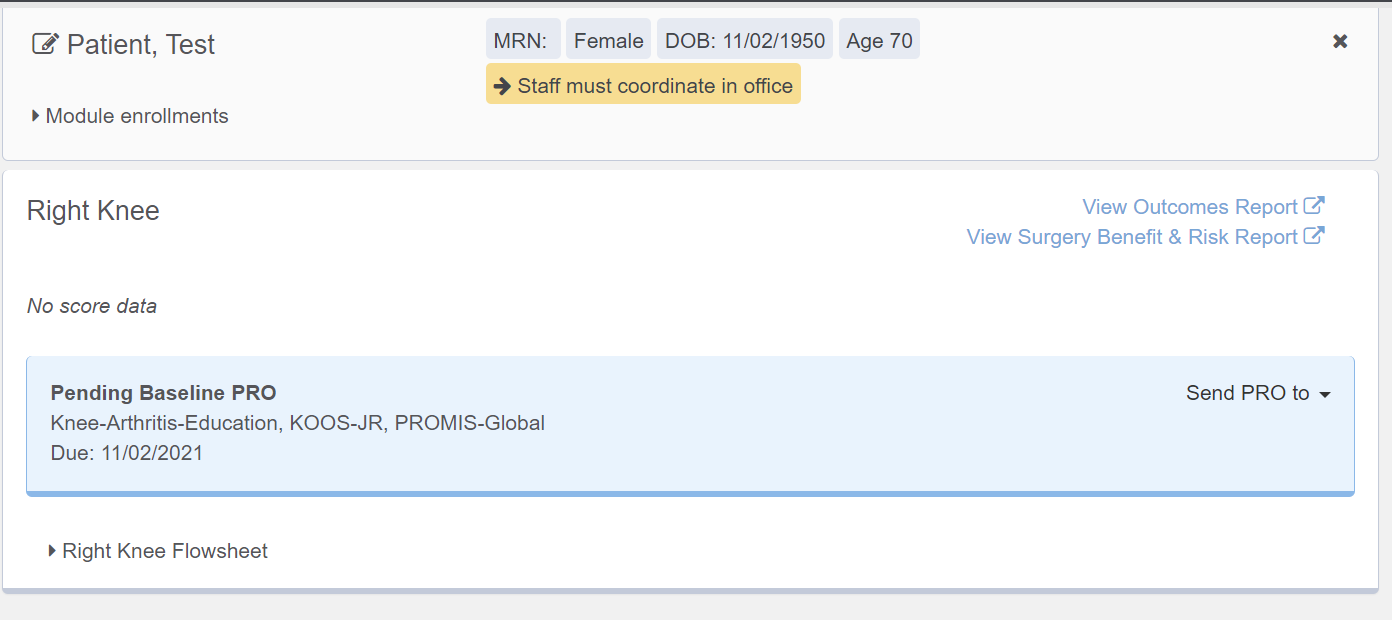


- - Click the dropdown menu “Send PRO to” and select “transfer to paper”


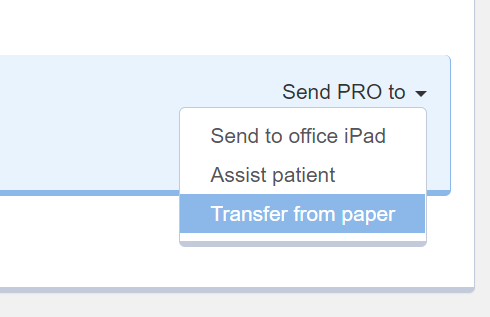


- - Enter the patient’s birth date and today’s date to open the module
  - The educational module will automatically appear, and the researcher will then hand the iPad to the patient for them to navigate through at their own speed. Researchers will use the following script:
    - *FULL TOOL:* “*This educational module should take about 5-10 minutes to read through, depending on your pace. When you are ready to move on to the next section, simply press “next” in the lower right-hand corner. When you reach the end of the education there will be a short quiz but do not worry, it is not graded. It is simply to help you test your own knowledge of what you learned. There will also be a few questions about your treatment preferences. Again, those are only for your benefit to help you think about the treatment options. There is no “right” answer. Finally, you will reach a set of survey questions identical to the ones you already answered in the lobby where you checked in. You may stop there, and I will fill out the survey using the information you have already given us. I will be back in 5-10 minutes and would be happy to answer any questions you have at that time.”*
    - *EDUCATION ONLY:* “*This educational module should take about 5-10 minutes to read through, depending on your pace. When you are ready to move on to the next section, simply press “next” in the lower right-hand corner. When you reach the end of the education there will be a short quiz but do not worry, it is not graded. It is simply to help you test your own knowledge of what you learned. I will be back in 5-10 minutes and would be happy to answer any questions you have at that time.”*
- Leave the room for 5-10 minutes
  - *Optional:* while patient is reading over the iPad materials, the RA completes the patient’s OM1 profile utilizing individual patient-reported outcome measure question responses to the KOOS, JR and PROMIS-10 Global as viewable in AthenaHealth.
  - The RA may also create a second module for patients reporting bilateral knee pain who are randomized to the full tool, so that the patient can get individualized scores for each side
- Return when time is up, and check if the patient has completed the educational module
  - If the patient is not done, politely offer more time
  - If the patient is done:
    - Close the tab with the educational module
    - *For education + preferences only patients:*
      - Thank them and leave the room
      - Inform the provider the patient is ready to be seen
    - *For full tool patients:*
      - Return to the OM1 enrollment tab and click “View Surgery Benefit & Risk Report”


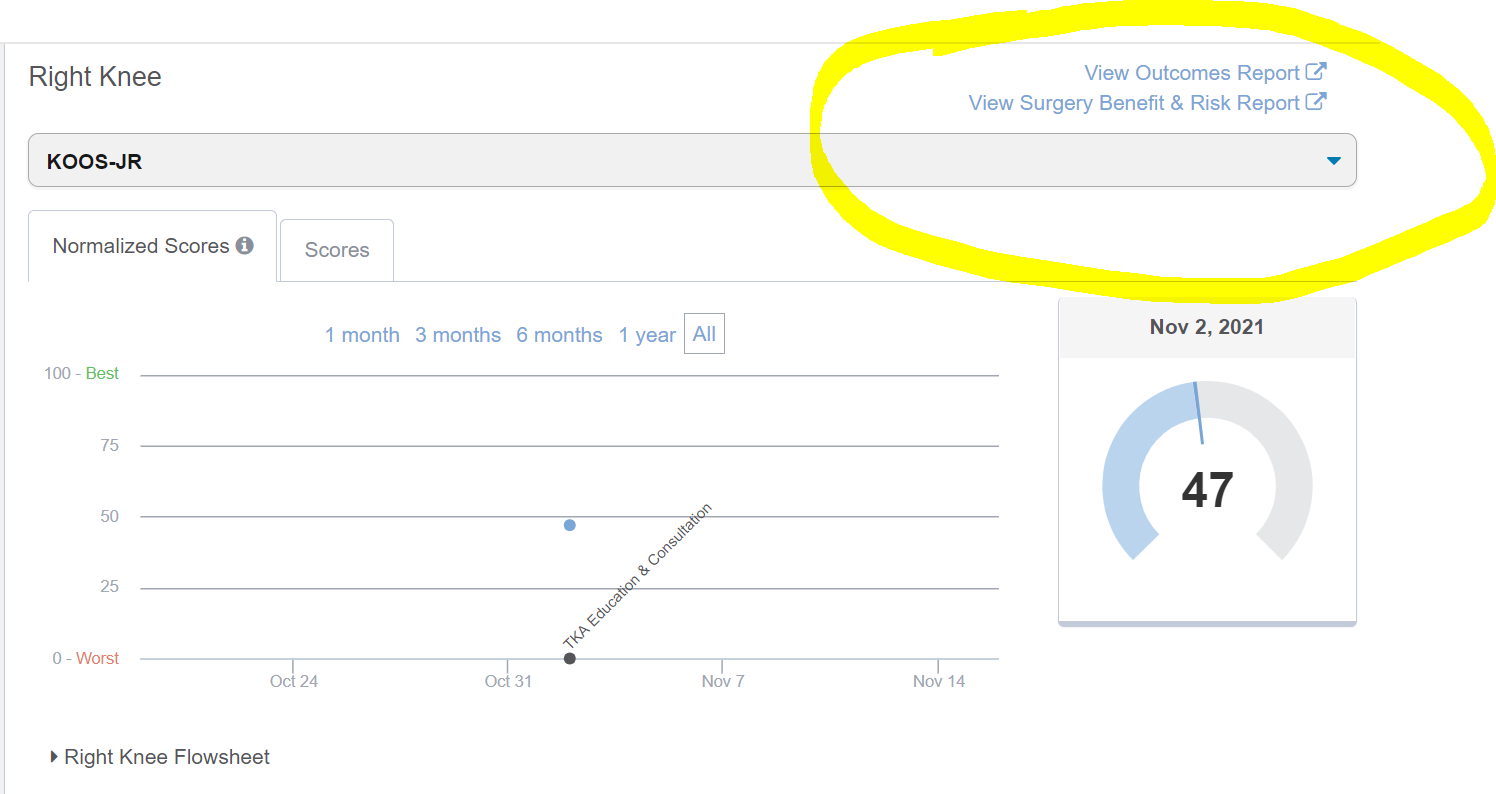


- - - - This will open a new tab with the Joint Insights report
      - Click on the “Select OA severity” dropdown menu to select the radiographic severity (KL 3 or 4)
      - Show the results with the patient and explain that they will receive a printed-out version for discussion with their provider
      - Leave the room
      - Print out the score report(s) and hand to the provider or hang in the basket on the exam room door
      - Inform the provider the patient is ready to be seen
      - Write the patient’s “Benefit” percentage under “notes” on the clinic whiteboard
- During the provider consultation
  - Research assistant must record for REDCap:
    - The number of minutes the counseling provider was in the room
      - This includes Dr. Bozic, Dr. Koenig, the Arthroplasty Fellow, Advanced Practice Providers (APPs).
      - As much as possible, the amount of time should only reflect time spent on the shared decision making conversation; this means that time spent on injections does not count, and time spent collecting medical history should not contribute either
      - However, we recognize there is variation in which provider or learner contributes to each part of the visit – for instance a learner (medical student or resident) might collect history for some patients which will *not* be included in the final time calculation, while the APPs might collect history for others and this *will* be included
      - Ultimately, we just record the exact number of minutes any of the above providers who are listed specifically by name spend in the room *except* after they come out to grab an injection and go back in to perform the injection
    - Enter the total consultation minutes into the appropriate field in REDCap
  - If the exact timing is missed, RA will confirm an estimate with the provider

***Recruitment Methods: Baseline Visit Questionnaires***

Patients from both randomization arms will be asked to complete the baseline surveys in REDCap at the end of their visit as soon as the consultation with their provider is complete:

- Patient Questions: Demographics
- Patient Questions: DQI
- Patient Questions: CollaboRATE
- Patient Questions: DCS10
- Patient Question: Satisfaction
- Patient-Facing Done Page (ask for patient preference of phone vs email for follow-up)

Researchers will then ask the relevant provider for the visit to complete the baseline survey in REDCap:

- Provider Question: Satisfaction

***Study Methods: Baseline Documentation***

The following steps must be completed by the researcher at the baseline visit to ensure proper documentation:

- On Box
  - Completion of the “Joint Insights Eligibility Tracker” as above
  - Completion of the “Joint Insights Follow-Up Tracker” for all enrolled patients
  - Completion of the “Joint Insights Dashboard” with total number of patients enrolled
- On REDCap
  - Completion of all baseline surveys (in “Event 1” column)
    - Complete “KOOS, JR” using Athena
    - Complete “PROMIS-10” using Athena
  - Mark which knee is primary in the 3-month “Follow-Up KOOS, JR” field
  - Mark which knee is primary in the 6-9 month “Follow-Up KOOS, JR” field
  - Schedule emails for the 3- and 6-9 month follow-up visits for patients preferring emails rather than phone calls
    - Schedule for a date 7 days before the 3-month follow-up date
    - Set up to 2 recurring reminders, each 7 days apart
    - Use the [dellmedsurgery@gmail.com](mailto:dellmedsurgery@gmail.com) email as the sender
    - Use the “Email Template” found on Box for the body of the email
      - Change [NAME] to the patient’s last name
      - Change [DATE] to the patient’s 3-month follow-up date

***Study Methods: Follow-Up***

Emails from REDCap will be sent automatically at 3 and 6 months after the baseline visit. We will send up to 3 emails (approximately 7 days apart) with a link to complete follow-up PROMs. If there is not response by approximately 3 days after the third email, a researcher will call the patient up to 3 times to complete the follow-up surveys via telephone.

- Follow-Up Surveys to complete at 3 and 6 months
  - KOOS JR
  - PROMIS Global 10
  - DRS
- Time Windows
  - 3 month follow-up window: 2.75 to 4 months after baseline visit.
  - 6-9 month follow-up window: 5.75 to 10 months after baseline visit.
- For patients for whom Spanish is indicated as the preferred language, the research assistant will call using the interpreter service.
  - To call the interpreter service:
    - Dial 1-855-886-2901
    - Enter 1755794
    - Enter MSKI PIN: 1115
    - Select Language: Spanish
    - Connect & conduct follow-up questionnaires using interpreting service

***Study Methods: Follow-Up Documentation***

- On Box
  - Completion of “Joint Insights Follow-Up Tracker” with the date of each follow-up email or phone call attempt
- On REDCap
  - Completion of relevant surveys for each patient at each appropriate time point
    - Patients contacted by email or text will complete surveys themselves
    - For patients contacted by phone, the researcher will enter the patient’s responses into REDCap
  - If patients decide to withdraw, they should be marked as “Withdrawn” in REDCap
  - If patients are lost to follow-up as indicated in the guidelines above, their follow-up surveys will be left blank in REDCap

***Reasons for Ineligibility or Withdrawal***

Patients are ineligible for the study if any of the following comes to light at any point during the baseline visit or follow-up visits:

- Patient declined
- Screening failure
- Patient withdrawal
- PI withdrawal of patient
  - Reasons:
    - Patient or provider determination that pt. does not have OA
    - Patient had a prior TKA or prior discussion with orthopaedic surgeon about TKA
    - Patient was being considered for revision joint replacement
    - Patient seeking care for trauma condition
    - Non-English or Non-Spanish speakers
    - Patients with BMI below 20 or above 46
    - Age under 45 or over 89
    - Participant unable to give informed consent / issue with cognitive status
    - Other

All patients withdrawing from the study must be marked as “Lost to Follow-Up” in REDCap.

**Data Management & Analysis**

Data collection at baseline and follow-up will be conducted as above in “Methods” section.

Data management will be conducted using REDCap as detailed above. This is a confidential and secure database protected on UT servers.

Formal comparative analysis will follow the intent-to-treat principle. Primary analysis will compare the intervention and control groups by using multiple linear regression analysis. The model will include DQI score as the response variable and, as explanatory variables, a binary indicator for the intervention group and 7 binary indicator variables representing the 8 strata in order to reflect the stratified randomization design. Additionally, as a secondary analysis, we will compare treatment decisions between the intervention and control groups by using multiple logistic regression. The model will include the treatment decision as the binary response variable and the same explanatory variables as in the linear regression model. Depending on the uptake of the intervention, additional analyses will follow the “per-protocol” principle wherein the main treatment variable will be whether the Joint Insights tool was used.

For analysis of the 3- and 6-9 month data, we will fit linear mixed models for continuous outcomes35 and generalized estimating equations logistic regression models for binary outcomes36, including indicator variables for time point, for treatment group, and for the interaction between the two (yielding treatment effects at 3 months and at 6-9 months). Owing to the balanced design, it will be possible to fit an unstructured correlation model to eliminate any sensitivity to correlation model misspecification.

Data monitoring is not required for this study. No interim analysis is planned.

This is a minimal-risk study. Although we don’t anticipate any adverse events, any adverse events will be reported to the local IRB.

The datasets used and/or analyzed during the current study are available from the corresponding author on reasonable request.

**Dissemination**

Planned publications:

1. Protocol paper
2. Results paper

Plan to follow ICMJE guidelines to determine authorship eligibility.

**Disclosures**

Dr. Jayakumar has received personal fees from Johnson & Johnson Medical Devices and the Purchaser Business Group on Health. Dr. Bozic has received personal fees from the CMS and Purchaser Business Group on Health; has stock options from Carrum Health; and has a leadership role with AAOS. Dr. Bozic has royalty agreements with Wolters Kluwer and Slack Incorporated. Dr. Bozic and Dr. Jayakumar are co-developers of the Joint Insights tool; they have no personal financial interest in the tool. The University of Texas at Austin has a royalty agreement with OM1, Inc. Dr. Tsevat receives royalties from Wolters Kluwer.

**Appendix**

***Patient FAQs***

Ask at various points if there are any further questions. Some FAQ from patients are:

- Will I have to make any additional trips for the purposes of this study?
  - No, everything that is not conducted on the day of your appointment will be conducted either over the phone or via email.
- Will this involve any experimental procedures or drugs?
  - No, the only experimental part of this study consists of whether you receive just educational materials or educational materials plus a risk:benefit report.
- How long will this take?
  - The total commitment time for the study will be approximately 1 hour over a period of 6 months. In addition to the time spent reviewing the educational materials and risk/benefit report today, we will contact you 3 months from now and 6 months from now to complete a few more surveys. Participation will only take about 10 minutes at each of those timepoints.
- How will this affect my appointment?
  - Your appointment will be conducted in the usual manner. This study will not affect the doctor’s or medical team’s actions.
- Will my information be released to insurance companies?
  - No, your information will remain private.
- Will I be compensated for my time and how so?
  - If you complete all three study timepoints (today, 3 month survey and 6-9 month survey), we will email or mail you a $25 gift card of your choice.
